# Effect of Washing Treatment on the Textural Properties and Bioactivity of Silica/Chitosan/TCP Xerogels for Bone Regeneration

**DOI:** 10.3390/ijms22158321

**Published:** 2021-08-02

**Authors:** Antonio Pérez-Moreno, María Virtudes Reyes-Peces, José Ignacio Vilches-Pérez, Rafael Fernández-Montesinos, Gonzalo Pinaglia-Tobaruela, Mercedes Salido, Nicolás de la Rosa-Fox, Manuel Piñero

**Affiliations:** 1 Instituto de Investigación e Innovación Biomédica de Cádiz (INIBICA), 11009 Cádiz, Spain; Antonio.perezmoreno@alum.uca.es (A.P.-M.); drvilches@dentalcamposoto.es (J.I.V.-P.); Rafael.montesinos@uca.es (R.F.-M.); Gonzalo.pinaglia@uca.es (G.P.-T.); Mercedes.salido@uca.es (M.S.); 2 Department of Condensed Matter Physics, Faculty of Science, University of Cadiz, 11510 Puerto Real, Spain; Maria.reyes@uca.es (M.V.R.-P.); nicolas.rosafox@uca.es (N.d.l.R.-F.); 3 Instituto de Microscopía Electrónica y Materiales (IMEYMAT), University of Cadiz, 11510 Cádiz, Spain; 4 Department of Histology, SCIBM, Faculty of Medicine, University of Cadiz, 11004 Cádiz, Spain

**Keywords:** hybrid xerogels, chitosan, tricalcium phosphate, N_2_ physisorption, t-plot, bioactivity, biodegradation, bone tissue engineering, osteoblasts, focal adhesions

## Abstract

Silica/biopolymer hydrogel-based materials constitute very attractive platforms for various emerging biomedical applications, particularly for bone repair. The incorporation of calcium phosphates in the hybrid network allows for designing implants with interesting biological properties. Here, we introduce a synthesis procedure for obtaining silica–chitosan (CS)–tricalcium phosphate (TCP) xerogels, with CS nominal content varying from 4 to 40 wt.% and 10 to 20 wt.% TCP. Samples were obtained using the sol-gel process assisted with ultrasound probe, and the influence of ethanol or water as washing solvents on surface area, micro- and mesopore volume, and average pore size were examined in order to optimize their textural properties. Three washing solutions with different soaking conditions were tested: 1 or 7 days in absolute ethanol and 30 days in distilled water, resulting in E1, E7, and W30 washing series, respectively. Soaked samples were eventually dried by evaporative drying at air ambient pressure, and the formation of interpenetrated hybrid structures was suggested by Fourier transformed infrared (FTIR) spectroscopy. In addition the impact that both washing solvent and TCP content have on the biodegradation, in vitro bioactivity and osteoconduction of xerogels were explored. It was found that calcium and phosphate-containing ethanol-washed xerogels presented in vitro release of calcium (2–12 mg/L) and silicon ions (~60–75 mg/L) after one week of soaking in phosphate-buffered saline (PBS), as revealed by inductive coupled plasma (ICP) spectroscopy analysis. However, only the release of silicon was detected for water-washed samples. Besides, all the samples exhibited in vitro bioactivity in simulated body fluid (SBF), as well as enhanced in vitro cell growth and also significant focal adhesion development and maturation.

## 1. Introduction

Polymer silica hybrid gels featuring both bioactivity and osteoconductive properties have experienced increasing research attention in recent years as biomaterials for bone tissue engineering [1,2,3,4]. In this sense, a broad range of organic polymers and biopolymers has been contemplated for the preparation of new hybrid silica gels, including collagen [5], gelatin [6], poly e-caprolactone [7], and also polysaccharides such as alginate [8], cellulose [9], and chitosan [10]. Among these organics chitosan (CS), a linear polysaccharide derived of chitin, composed of glucosamine and N-acetylglucosamine, has stimulated scientific research interest in the last two decades to investigate its use in biomedical applications [11,12,13]. The amine and hydroxyl groups in chitosan facilitates the hybridization via sol gel with the silica network, mainly by condensation with silanol groups. As a result, the polysaccharide strongly binds silicates by their hydroxyl groups and exhibits a large fraction of silanol groups bearing Q3 species, although a large fraction of the silicon atoms is completely crosslinked by siloxane bonds, as described by Watzke et al. [14], which supposes one of the first approaches for the synthesis of these hybrid materials. In the field of biomedical engineering, SiO_2_/CS hybrids have shown its effectiveness in several interesting applications, e.g., to support cells adhesion and growth [15], to improve biomineralization in metallic implants [16], and as drug delivery systems [17]. In case of SiO_2_/CS hybrids incorporating cross-linking agents, such as 3-Glycidyloxypropyltrimethoxysilane (GPTMS) [18,19] or genipin [20], and carbon nanotubes (CNTs) [21], the biomedical applications are widespread, and various studies to expand cell proliferation [22] or to promote bone regeneration as scaffold [23] have been accomplished. 

Natural polymers, as described in this paper, have attractive properties for the construction of 3D second generation scaffolds, such as biocompatibility and biodegradability. The mechanical and osteoconductive properties of these polymers can be enhanced by producing composites with bioactive ceramics, and the biomineralization can be tailored with bioactive elements such as chitosan, looking for interfaces, which would elicit a specific biological response (i.e., osteoconduction) to improve osseointegration [24,25,26,27] A critical mechanism for cellular interaction with the extracellular matrix, and with biomaterials, is the process of cell adhesion. One of the key pieces in the subsequent mechano-transduction process appears to be the so-called focal adhesions [28]. In addition to facilitating cellular tethering to the scaffold, focal adhesions form the basis of filopodia exploration and the subsequent lamellipodia ruffling and cellular spreading in response to topographical cues. During the cell–material interaction, ionic dissolution from the hybrid biomaterial surface plays a significant role. Additionally, in the course of biomineralization, surface silanols may act as nucleation sites for apatite deposition, leading to the formation of biomimetic hydroxyapatite (HAp) layer [29]. Moreover, several studies on mesoporous silica-based materials, containing different amount of calcium phosphates (CaPs), have reported that the release of Ca and Si ions from the material leads to a faster supersaturation of a simulated body fluid (SBF) solution, around the porous surface [30]. These changes in ionic concentrations in SBF promote and accelerate the development of a bioactive apatite-like layer, which might be advantageous to osteoblasts responses [31].

One of the most studied CaPs, regarding its use as basic material for bone repair, is tricalcium phosphate (TCP; Ca_3_(PO4)_2_, with a Ca/P ratio of 1.5. Three polymorphs correspond to this composition: β-TCP (low temperature), α-TCP (metastable at room temperature) [9,10], and α’-TCP (high temperature) [32]. Additionally, the α-phase is the most reactive in aqueous system, among others (CPCs) [33]. As a consequence, a growing interest in α-TCP as a biomaterial for bone implants has arisen, dealing with the capability of a-TCP to be replaced by new bone [34].

Sol-gel methods offer several advantages over conventional processing technologies and an excellent way of obtaining hybrid biomaterials for biomedical applications [4,35,36]. Accordingly, the technique involves six steps, in the following sequential order: hydrolysis and polycondensation, gelation, aging, washing, and drying. The washing step has great influence on the textural properties of silica-based xerogels, such as surface area, pore volume, and pore size distribution, as well as xerogel shrinkage and resulting shape. Ethanol excess leads to partial or complete removal of water out of pore volume and subsequent adsorption of ethanol onto silanol groups. These reactions accelerate condensation and inhibit polymerization, thus eliminating micropores and preventing densification of silica skeleton accordingly. Consequently, when compared to water-washed xerogels, ethanol-washed xerogels displayed enhancement of textural properties with increasing total surface and pore volume (e.g., 1000 m^2^·g^−1^ and 1.5 cm^3^·g^−1^ have been measured from ethanol-washed sodium silicate-derived xerogels [37]). Additionally, textural properties could be affected by several other factors during gel preparation and post-processing, e.g., silica source, pH, temperature, aging, and washing soaking period.

Numerous studies have been focused on the preparation via sol gel of SiO_2_/CS hybrids using tetraethoxysilane (TEOS) as silica source, mostly reporting interpenetrating network hybrid coatings or membranes of silica and CS [38,39]. In addition, the synthesis of SiO_2_/TCP has been also addressed [40,41] as well as CS/TCP for bone cements [42]. The combination of CS and gelatin biopolymers with β-TCP scaffolds for BTE has been described as well [43]. Nevertheless, despite significant achievements in the preparation and characterization of many types of hybrids for biomedical purposes, and the hundreds of articles related to this topic, more efforts are needed to successfully address potential applications of micro-mesoporous xerogels combining hybrid networks with CaPs, as biomaterials for tissue engineering.

The aim of this work was to synthesize silica/CS/TCP hybrid xerogels by sol gel, using tetraethoxysilane (TEOS), chitosan (CS) powder, and TCP powder, intending to improve “in vitro” biomineralization and osteoblasts responses. In addition, this work explores the effect of ethanol and water as washing solvents on textural properties of the obtained xerogels, particularly the specific surface area, micro and mesopore volume, and pore size. Likewise, this work investigates the biomineralization and biodegradation responses in saline solution and addresses an in vitro study as an initial requirement for the designed xerogels to be proposed for clinical use. The results obtained with the biomaterials here described appear to induce in vitro positive changes on human normal osteoblasts in the direction of an adequate osteoblasts differentiation with optimal cell adhesion. Furthermore, the biomaterials described can be easily sterilized under standard clinical protocols, a princeps key factor for clinical use. Several steps are needed, anyway, until the materials described herein could be clinically applied.

## 2. Results and Discussion

### 2.1. Synthesis of Silica/CS/TCP Xerogels

Silica xerogels with varied CS nominal contents (SCSx; x = 0, 4, 8, 16, 20, 40 CS wt.%) were obtained via sol gel from the hydrolysis of tetralkoxysilane (TEOS) and copolymerized with CS biopolymer in acid media. TCP was added to TEOS/CS sol with a CS 8 wt.% fixed composition to obtain SCS8Ty (y = 10, 20 TCP wt.%) samples. For the inorganic tetraethoxysilane, the H_2_O/TEOS molar hydrolysis ratio (R_w_) equals 30, thus allowing a highly crosslinked network. A comprehensive picture with the scheme of the synthesis procedure is given in Section 3.2, including a description of post-gelation treatment. In brief, once gelation and aging steps are terminated, three different washing procedures were designed as follows: washing in absolute ethanol for (i) 1 day, (ii) 7 days, and (iii) washing in distilled water for 30 days. Afterwards, samples were dried at 80 °C in ambient air pressure, and the resulting sample washing series were labelled as:= E1, E7, and W30, respectively. An additional sample series of unwashed (U) xerogels, directly dried with no treatment after gelation and aging, was added for comparison. Table 1 lists the results of the C and N elemental analysis (EA) of the different xerogel sample series.

An increase in C/N ratio observed for E1 and E7 ethanol-washed samples, respect to U ones, is associated with soaking time in the washing solvent. The calculated CS amount, in all cases, was lower than the expected nominal content, being undetectable in W30 xerogels. The same conduct has been previously reported elsewhere by us, even using a cross-linker in the process [44], and others [45]. These results indicate a considerable dissolution and release of CS, and as a consequence, the final composition of the hybrids did not match the nominal content. Nevertheless, as shown in Table 1, it was possible to obtain and characterize a variation in CS concentration in the studied xerogels and evaluate its influence on the physical and structural properties. However, in this article, the abovementioned nominal contents will be used as reference in sample code for both CS (and TCP), in order to simplify the presentation and discussion of results.

### 2.2. Physical Characterization and Structural Properties

#### 2.2.1. SiO_2_/CS Xerogels

The specimens were all crack-free after drying, with cylinder shape 10–15 mm height and 3–7 mm diameter and resistant to physical touch. This point validates the procedure of hydrolyzing the alcoxide at a molar ratio as high as Rw = 30, in acid media, as an effective strategy to synthesize crack-free monolithic xerogels [46,47]. Samples were also visually homogeneous throughout the volume except for unwashed samples, which presented an heterogeneous incorporation and distribution of CS, especially for higher CS contents, as shown in Figure S1 of the Supplementary Materials. This could be the sign of the development of class I hybrid organic/inorganic structures, where organic and inorganic components are embedded, suggesting the formation of interpenetrating a hybrid polymer between SiO_2_ and CS networks [48].

**Figure 1:**
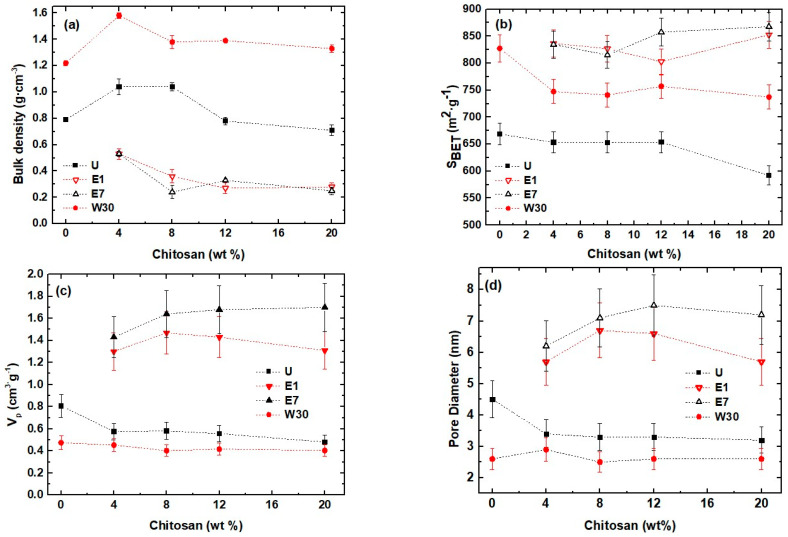
(**a**) Bulk density; (**b**) BET surface specific area (S_BET_); (**c**) pore volume (V_p_), and (**d**) pore diameter, for SiO_2_/CS xerogels as a function of the CS content.

The influence of CS nominal content on bulk density and textural properties for all the SCSx samples was investigated, and the results are shown in Figure 1, and as can be deduced, the gradual amount increase in CS in the silica porous matrix was responsible for only small variations with regard to bulk density and textural properties for each particular washing set.

The textural features of the samples were investigated by N_2_ physisorption experiments. In Figure 1b–d, the BET-specific surface (S_BET_), specific pore volume (V_p_), and pore size (D) are presented as a function of the CS content, respectively (see Table S1 of the Supplementary Materials for more details). In general, E1 and E7 samples displayed, as expected, very similar behavior between them, with CS increase, and also better textural properties than U and W30 samples. Relatively low V_p_ and D observed values in U xerogels must be attributed to the presence of unreacted residues, which are filling and partially blocking the pores of U xerogels.

Variations observed in densities or textural parameters in E1, E7, and W30 can be related to pore coalescence structural processes taking place during evaporative drying, in case of water-washed xerogels. This should be due to the proliferation of silanol groups while immersed, which provokes the acceleration of silanol condensation, leading to micropore creation and also pore volume reduction. This behavior was also observed by Davis et al. [49], who indicated that the pore structure of silica gels may be considerably altered during the aging process by water solvent, and that their wet gel features may be partially preserved upon drying. Meanwhile, in comparison with W30, E1 and E7 (ethanol-washed) samples experimented an increase in pore volume and decrease in microporosity, which indicates that ethanol washing could produce a further aging process compared to water-washing according to Park et al. [37] and Fidalgo et al. [50], who previously studied the influence of different washing solvents on textural properties of silica-based xerogels. 

#### 2.2.2. SiO_2_/CS/TCP Xerogels

With the intention to study the influence of TCP content on SiO_2_/CS xerogels, a representative SCS8 sample was selected, so as to evaluate the effect of incorporating 10 and 20 wt.% TCP on the further biomineralization of these materials. The bulk density and textural properties of SCS8, SCS8T10, and SCS8T20 xerogels were also measured, and the results obtained are listed in Table 2.

In general, the inclusion of TCP in the SiO_2_/CS porous matrix provoked an important increase in the density up to 40–50% upon increasing TCP content, except for W30 xerogels, as shown in Table 2, which experienced only a 4–7% increase. This effect was attributed to the release of both CS and TCP during soaking in water. On the other hand, variations in densities and textural data, between the unwashed and E1, E7, and W30 washing series, showed similar trends than the observed in SiO_2_/CS xerogels.

The results from Figure 1b and Table 2 reveal high specific surface areas, especially in ethanol-washed samples, that compare to those of similar silica/chitosan hybrid aerogels, meaning that their structures are highly crosslinked and interconnected, as reported by Ayers et al. [51], Buckley et al. [52], and Perez-Moreno et al. [53]. Additionally, it can be stated that soaking in water causes structural changes in the hybrid gel network, as deduced from the textural data observed in W30 samples. As a consequence, unlike ethanol, water-washing leads to a greater gel volume shrinkage, thus leading to high bulk densities. Besides, the small pore-specific volumes and pore sizes, found at the down limit of the mesopore domain, indicate a contribution of micropore size distribution for W30 xerogels.

**Figure 2:**
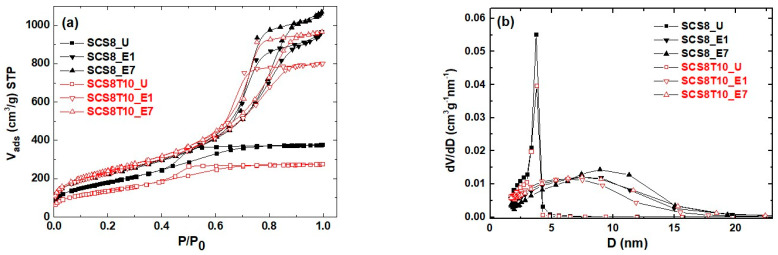
(**a**) N_2_ isotherms and (**b**) pore size distribution of SCS8 and SCS8T10 xerogels subjected during sol-gel processing to different washing treatments (U, E1, and E7) before evaporative drying.

A comparison of nitrogen isotherms for SCS8 and SCS8T10 (U, E1, and E7) xerogels and their corresponding pore size distribution (PSD) are shown in Figure 2. This selection is, under our consideration, representative for giving a general description of the structural properties of these sample series.

According to IUPAC classification, the N_2_ adsorption–desorption isotherms showed in Figure 2a were all of type IV, characteristic of mesoporous materials with H1 hysteresis loop, characteristic of interconnected network of pores with a narrow pore size distribution and a high pore size uniformity [54]. Samples E1 and E7 presented very similar hysteresis loop, with a similar sharp step of the desorption branch at P/P_0_ values over 0.6. In its place, the hysteresis loop of U samples moves to a lower P/P_0_ (about 0.4), indicating a decrease in the average pore size of those samples. Their corresponding isotherms also present a desorption hysteresis and a well-defined horizontal plateau at relative pressure 0.6–1.0, with a delayed desorption branch much less steep than in case of washed samples. These results are indicative of complex evaporative processes, from ink-bottle-type pores, which cause pore blocking, mainly attributed to the presence of unreacted residues of CS and TCP [55]. The pore size distribution (PSD) (Figure 2b) showed that the pore volume is mostly found in the mesopore domain, with diameters ranging from 3 to 20 nm, while the t-plot analysis revealed the absence of micropores in all the samples specified in that PSD. The t-plot method allows us to determine the micropore volume and micropore area from the physisorption isotherm, without measuring low-pressure micropore-filling portion of the isotherm [56]. In comparison with U samples, E1 and E7 xerogels led to an increase in the surface area (ca. 120–240%) and pore volume (ca. 250–500%) (see Table 2), while their PSD show broader pore size distribution, with average pore size in the range of 4.7–7.1 nm. On the other hand, U xerogels showed relatively low surface areas (360.8–485.5 m^2^·g^−1^), due to the effect of Ca^2+^ as network modifier, which results in partial destruction of the silica network [35]. Unwashed samples exhibited the smaller average pore size (3.0–3.3 nm) and also displayed a very narrow distribution.

**Figure 3:**
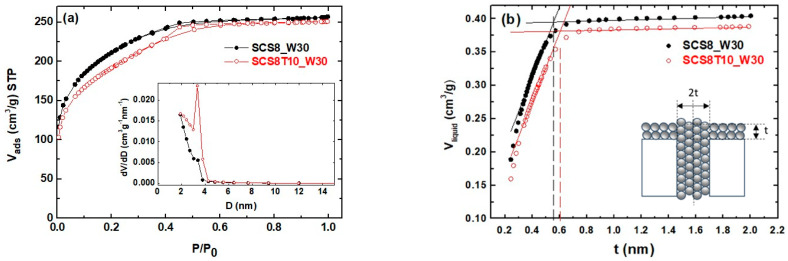
(**a**) N_2_ isotherms and PSD (inset) of SCS8_W30 and SCS8T10_W30 xerogels (**b**) t-plot curves indicating the half of the maximum pore width, as schematically shown in the inset [59]. Volume STP adsorbed nitrogen in the y-axis has been converted to the liquid equivalent one.

Instead, all the N_2_-isotherms from W30 xerogels (Figure 3a) are of type I, typical from solids with relatively small external surfaces that contain both wider micropores (1 nm) and narrow mesopores [54]. The corresponding PSD reaches the lower limit of the technique (2 nm), as shown in the inset. Furthermore, Figure 3b shows the resulting t-plot curves, where the volume of nitrogen adsorbed vs. t, the statistical thickness of an adsorbed layer on a nonporous reference surface at a corresponding relative pressure, is plotted. The t values were calculated from the experimental relative pressures used in obtaining the adsorption data, from the equation proposed initially by DeBoer [57] (see Table S2). In the absence of micropores, a line through the origin or slightly negative will result in the extrapolation of the t-plot to the y-axis. In this case, the calculated micropore volume and area will be zero. Otherwise, the t-plot will exhibit a positive intercept when micropores are present. By transforming the adsorbed nitrogen gas to liquid equivalent ones in y-axis volume, the resultant intercept of the t-plot directly gives the micropore volume [58].

In Figure 3a, the isotherms are concave to the P/P_0_ axis and show a long horizontal plateau up to high P/P0 values, with a small desorption H1 hysteresis loop in the desorption branch, due to capillary condensation in small mesopores [60]. The steep uptake at very low relative pressure is due to enhanced adsorbent–adsorptive interactions in narrow micropores, resulting in micropore filling, while the curve increase at low pressures is related to the high surface areas values calculated (663.7–741.0 m^2^·g^−1^ from the BET method). These W30 samples exhibited the smaller pore volume (0.39–0.42 cm^3^·g^−1^) and average pore diameter (2.5–2.7 nm) from the Barrett, Joyner, and Halenda (BJH) method (see Table 2) of all studied samples. The corresponding t-plot curves are shown in Figure 3b, exhibiting two linear regimes, the first one prior to the condensation step due to mesopore filling and the second one after, due to the adsorption on the external surface. For the analysis, the intercept of the first linear segment gives the volume of micropores (V_micro_), and the corresponding slope is equivalent to the total surface (S_tot_), which for a nonporous adsorbent, it equals the BET surface. At higher P/P_0_, above mesopore filling, the second linear regime slope gives external surface (S_Ext_) (S_Ext_ = 1000 × slope), and its corresponding intercept gives the total pore volume (V_Tot_). The surface and the volume of the mesopores are simply given by the following equations [57,61,62]:

S_meso_ = S_tot_ − S_ext_ (1)

V_meso_ = V_tot_ − V_mic_ (2)

Table 3 lists the t-plot parameters obtained from SCS8_W30, SCS8T10_W30, and SCS8T20_W30 xerogels, corroborating the existence of a fraction of micropores in all three samples. The results of t-plot application to the rest of the W30 xerogel compositions can be found in Table S2 (in the Supplementary Materials). For statistical purposes, all the results obtained were averaged over at least three independent runs, to check reproducibility. The data analysis confirmed the presence of micropores in W30 xerogels, in agreement with the previously reported data (see Figure 1a). Thus, the use of water as washing solvent induced pore restructuring in xerogels, decreasing average pore size and pore volume towards micropore domain. This effect was attributed to the partial collapse of pores due to silanol condensation developed during the water soaking step, in agreement with Dollimore et al. who described the phenomena in terms of movement of silica from large to small pores and to the external surface [63].

### 2.3. Thermal Characterization

**Figure 4:**
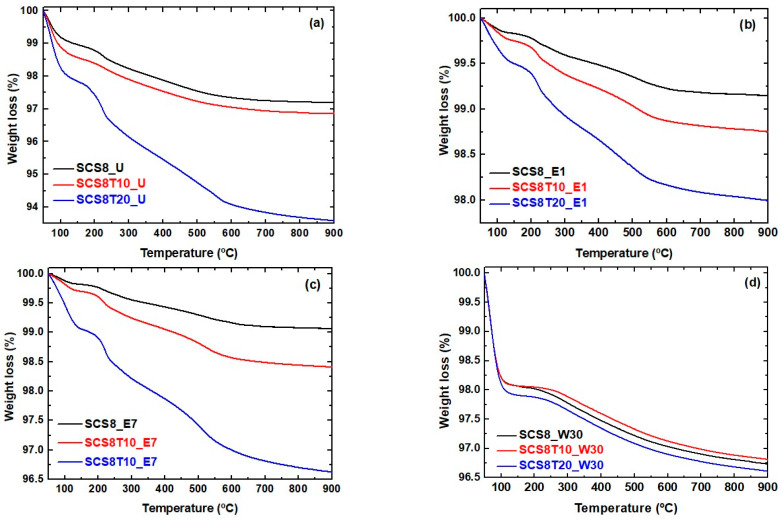
Thermograms of SCS8, SCS8T10, and SCS8T20 xerogels dried after four different washing procedures (**a**) U; (**b**) E1; (**c**) E7; (**d**) W30.

Thermograms of the SCS8, SCS8T10, and SCS8T20 xerogel are shown in Figure 4, for U, E1, E7, and W30 washing treatments. The experiments were performed under air atmosphere from 50 °C up to 900 °C. Some similarities and differences are apparent by comparing thermal profiles between the four-sample series.

As a first remark, a total weight loss in the range 2.5–6.5% was observed for the unwashed xerogels, which were higher than the 0.75–2% observed for E1 and 1–3.7% for E7, while the losses for W30 xerogels were found in a narrow range above 3.5%. In all cases, an initial weight loss of about 1–2 wt.% from 50 °C to 100 °C takes place, associated with the evaporation of physically trapped water, thus confirming the hydrophilic character of the xerogel surfaces. Additionally, the thermal decomposition profiles of U, E1, and E7 in Figure 4a–c, respectively, were very similar, and the same thermal events should be implicated, e.g., SCS8 and SCS8T10 thermal profiles of these washing series show two main step losses after the evaporation of water: the first one in the range 120–250 °C, accounting for the removal of silanols and combustion of CS, and furthermore, a gradual weight loss from 260 to 700 °C, accounting for dehydroxilation of isolated -OH groups [53]. Otherwise, the thermal profile of SCS8T20_U indicates the influence of TCP, giving an explanation for the step loss (ca.1.3 wt.%) occurring in the range 160–210 °C, presumed to be due to a dehydration of a crystalline phase of TCP, according to Jinlong et al. [64]. The following observed step loss (ca. 2.6 wt.%; 240–600 °C) is due to a new dehydration of crystalline calcium phosphate, probably leading to a residue of pyrophosphate [64]. This weight loss develops together with dehydroxilation of the silica and combustion of CS in SCS8T20_U. A final weight loss (ca. 0.5 wt.%), from 600 °C to the end of thermal scanning, is due to thermal degradation of organic residues.

The amount of weight losses related to the evaporation of physically adsorbed water varied between the three washing series in the following order: W30 > E7 > E1, meaning that the surface hydrophilicity of xerogels increases with increasing the soaking period of their respective washing treatment. Conversely, the thermal decomposition measured for the three W30 studied samples (Figure 4d) exhibits almost identical thermal behavior between them, displaying only two weight losses: first (ca. 2 wt.%; 50–100 °C due to evaporation of physically adsorbed water and second (ca. 1.50 wt.%; 170–700 °C), attributed to dehydroxilation of silanols, at a maximum decomposition temperature of 300 °C, thus presenting a similar behavior of a pure silica xerogel [65].

### 2.4. FTIR Spectral Analysis

**Figure 5:**
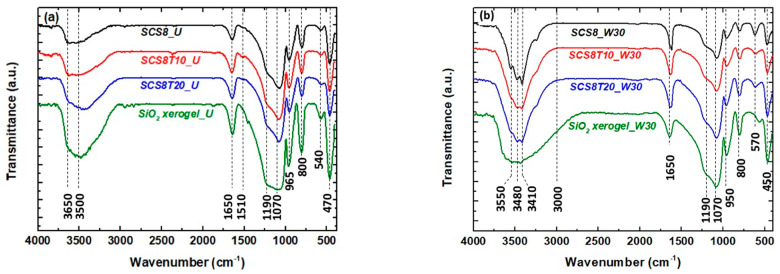
FTIR spectra of SCS8, SCS8T10, SCS8T20, and pure SiO_2_ xerogels: (**a**) U; (**b**) W30.

Figure 5a shows infrared absorption spectra of SCS8, SCS8T10, and SCS8T20 for U and Figure 5b for W30 hybrid xerogels. Although the principal bands of silica were well determined, the overlapping with bands from CS or TCP avoided a clear identification of both components.

The ten major peaks observed in Figure 5a corresponding to the following wavenumbers, 470, 540, 800, 965, 1070, 1190, 1510, 1650, 3500, and 3650 cm^−1^, are associated with the following absorption bands: 470 cm^−1^ and 540 cm^−1^, related to bending vibrations and Si–O− rocking mode, respectively [53]. At a higher frequency, absorption bands at 800 cm^−1^ are related to symmetric Si–O–Si stretching vibrations, and bands at 965 cm^−1^ to surface silanol Si–O(H) bond stretching [66]. The band at 1070 cm^−1^ and its high frequency shoulder at 1190 cm^−1^ are due to asymmetrical vibrations of longitudinal stretching of Si–O–Si. Besides, in acidic conditions, chitosan has two absorption bands: one at 1650 cm^−1^ due to C=O stretch of the secondary amide in acetylated units, which overlaps with vibration signal from water bending, while the other band can be found at 1510 cm^−1^, from the N–H bond of the primary amine in deacetylated units [67,68], and can be hardly discerned for the spectra of U sample series. Vibrations in the region of 3000–3750 cm^−1^ are attributed to hydroxyl groups O–H stretching modes and also to residual water. In this region, a characteristic band between 3400 and 3200 cm^−1^ due to hydrogen bonding interaction between the carbonyl group of chitosan and the silica network occurs for U samples. The resolution of this broad band into two peaks at 3410 and 3480 cm^−1^, exclusively observed in W30 xerogels, (Figure 5b) is assigned to surface –OH isolated, germinal, and vicinal silanol groups [53]. This Si–OH surface film accounts for the increase in the hydrophilic character of the surface of these materials, as well as for the siloxane ring structure, regarding the micro-mesoporosity [69,70]. Additionally, the shoulder at about 3550 cm^−1^ in these three samples is associated with the presence of ethanol from the washing procedure. Additionally, TCP shows intensive bands at 1121, 1083, 1045, 973, and 946 cm^−1^, as reported in the literature by Habelitz et al. [71], which correspond to the characteristic stretching modes of tetrahedral [PO_4_]^3−^ groups of TCP, which were probably overlapped or not detected in Figure 5a.

### 2.5. Biodegradation

**Figure 6:**
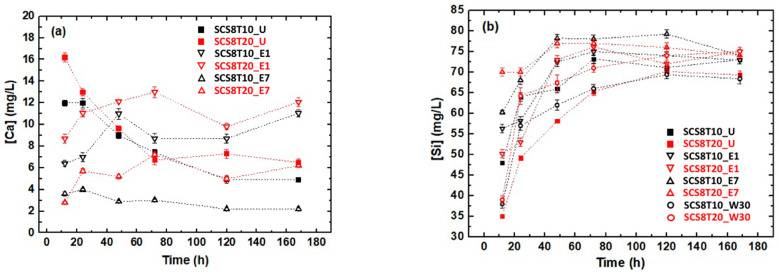
(**a**) Ca and (**b**) Si release from SCS8, SCS8T10, and SCS8T20 xerogels soaked in PBS and previously subjected to U, E1, E7, and W30 washing treatments before evaporative drying.

Samples were soaked for one week in PBS solution and the time evolution of calcium, and silicon release was examined for SCS8T10 and SCS8T20 samples that underwent U, E1, E7, or W30 washing treatments. The results obtained for Ca release are presented in Figure 6a, normally exhibiting a decreasing variation of [Ca] with soaking time in PBS for unwashed samples from above 16 mg/L to 5 mg/L. More precisely, ethanol-washed samples showed a decrease in calcium release with maximum 12 mg/L and minimum 2 mg/L, depending on the type of washing treatment they were subjected. In this sense, they can be well ordered as: SCS8T20_E1 > SCS8T10_E1 > SCS8T20_E7 > SCS8T10_E7 (see Figure 6a).

Besides, it has to be remarked that although SCS8T10_W30 and SCS8T20_W30 samples generated high Si concentrations (Figure 6b), they did not give any sign of liberation for Ca ions. Additionally, silicon release increases with soaking time in PBS in all cases, for ethanol- and water-washed samples, up to a constant value between 67 mg/L and 75 mg/L. The observed high initial silicon level (12 h soaking in PBS), 70 mg/L, for SCS8T20_E7 is attributed to the Si diffusion and release, due to the extent of degradation attained in the network. Same reasoning should be applied to explain the Si release for the rest of samples at the beginning of the experiment in Figure 6b.

Furthermore, as the biodegradation of the silica/TCP xerogels may be a combination of different processes implying corrosion, fracture, disintegration, and chemical dissolution, they can lead not only to increases in Ca, P, and Si locally at the surface, but also to changes in pH [72]. For that reason, the time evolution of pH of several U and W30 xerogels soaked in PBS (2 mg/mL) was checked, and the results obtained can be seen in Figure S2 (in the Supplementary Materials). It was observed that for both types of samples, the pH of the PBS solution decreased after the first 12 h of soaking and remained almost unchanged for 7 days throughout the experiment. Accordingly, Figure S2a shows pH reductions from 7.4 (for the standard PBS solution) to 7.1 for SCS8_U or to 6.45 for SCS8T20_U, while corresponding SCS8_W30 and SCS8T20_W30 samples in PBS (Figure S2b) present pH values between 7.3 and 7.0. Both the extent of acidification and the rate of pH decrease were found to be more pronounced in cases of U samples compared to W30 samples, which behaves almost as pure SiO_2_ xerogels [73].

### 2.6. Biomineralization

**Figure 7:**
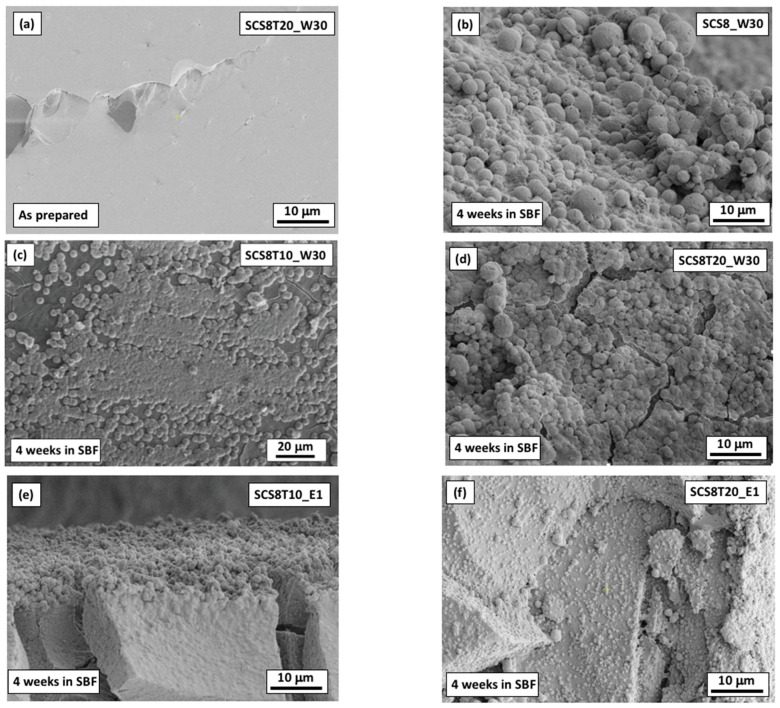

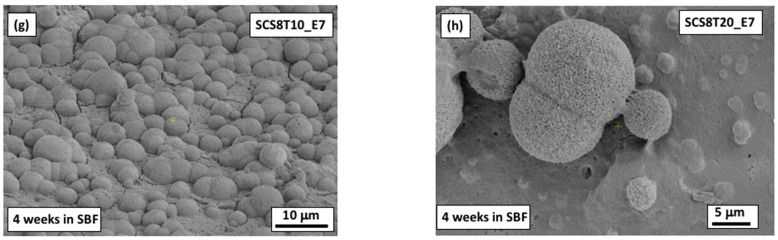
SEM micrographs of the apatite layer formed after 4 weeks soaking in SBF: (**a**) control (as prepared SCS8T20_W30 xerogel); (**b**) apatite layer on SCS8_W30; (**c**) different size crystals of apatite covering the surfaces of SCS8T10_W30; (**d**) SCS8T20_W30; (**e**) layer of homogenous apatite on SCS8T10_E1; (**f**) apatite crystals of different size growing and covering the surface of SCS8T20_E1; (**g**) apatite homogenous layer on SCS8T10_E7; (**h**) spherulites with different size and nucleation process of apatite from the surface of SCS8T20_E7 xerogel.

Figure 7 displays the scanning electron microscope (SEM) micrographs of the biomimetic HA layer formed on several xerogels with a fixed CS 8 wt.% content and 0, 10, and 20 wt.% TCP with four weeks soaking in SBF. The corresponding EDS analysis for Ca and P content were performed both before and after soaking in SBF for 28 days, and the results are presented in Table 4, showing a variation in Ca/P ratios from 1.37 to 2.25. According to SEM images, despite differences in processing and composition, it is clear that all xerogels exhibited a good in vitro bioactivity response. As SEM image control, the fracture surface of SCS8T10_W30 xerogel, before soaking in SBF, was taken. (Figure 7a). It reveals a flat homogeneous surface with a propagating crack-tip, as the only relevant feature. Figure 7b–h show the morphology of the grown layer of apatite on the surface of both SCS8T10 and SCS8T20 xerogels, subjected to W30, E1, and E7 washing procedures. All of these SEM photographs revealed the formation of micron and sub-micron spherulitic crystals of HA on the xerogel surfaces, which are almost totally covered, displaying average diameters in the range 1–5 μm, for samples (see Figure 7b–d, respectively). Likewise, E1 xerogels (Figure 7e,f, respectively) showed a layer of spherulites covering almost all their surfaces, whereas they displayed a great variety of diameters in case of SCS8T20_E1, from submicron to above 5 μm, indicating that heterogeneous HA nucleation was promoted at the surface of the xerogel. In addition, E7 xerogels show greater size spherulites on its surface, in the range 5–10 μm for SCS8T10_E7 (Figure 7g) and from 1 μm to 10 μm for SCS8T20_E7 (Figure 7h). Additionally, the last micrograph clearly shows some small spherulites growing from nucleating sites at the surface of the material.

By performing elemental mapping at the microstructural level by scanning electron microscopy (SEM) with energy dispersive X-ray spectrometry (EDS), the results confirmed the existence of high concentrations of Ca and P at the surface sites abovementioned, as shown in Figure S3 of the Supplementary Material.

Figure 7 also gives visual support about the mechanism of the biomineralization process, as it shows the intermediate and final stages of the formation of apatite, probably induced by surface functional groups composed of Si–OH, as detected by FTIR [29] (see Figure 4). The negative charge of OH– groups acts as preferential nucleation sites for calcium ions, which are attracted from SBF. This, in turn, attracts a phosphate group to create nucleation centers and the aggregation of nanosized clusters of calcium phosphates [74]. These processes were best observed for samples containing TCP (SCS8T20_E1 and SCS8T20_E7). As a consequence, the relationship of high Ca–P content in these xerogels with the improvement of heterogeneous nucleation of HA has been demonstrated (see Figure S3 of the Supplementary Materials).

Table 4 lists the results from EDS analysis for some of the studied xerogels after 4 weeks soaking in SBF and revealed different characteristics stages of the hydroxyapatite (HA) formation, whose Ca/P atomic ratio, considered as a reference, was 1.67. The surfaces sites (see Figure 7) with increasing Ca/P ratio up to 2.25 are probably found in a first stage of HA formation, in which the HA surface increased to form an amorphous phase of Ca-rich calcium phosphate. Meanwhile, decreasing Ca/P values up to 1.37 are frequently associated with the existence of Ca-poor calcium phosphate amorphous phases. Ca/P ratios approaching 1.67 (e.g., 1.58 for SC8T20_E1) are close to subsequently producing the biomimetic crystallite of apatite, growing in complex crystal spherulite assemblies, with further increasing immersion times [29,55,74]. Besides, SCS8T10_U and SCS8T10_W30 surfaces were also analyzed by EDS before SBF soaking. It was observed that the unwashed sample, unlike the water-washed xerogel, gave a Ca/P ratio of 1.37 indicative of the presence of TCP. All these findings confirm the results obtained from elemental analysis (see Table 1).

### 2.7. Osteoblast Behavior

**Figure 8:**
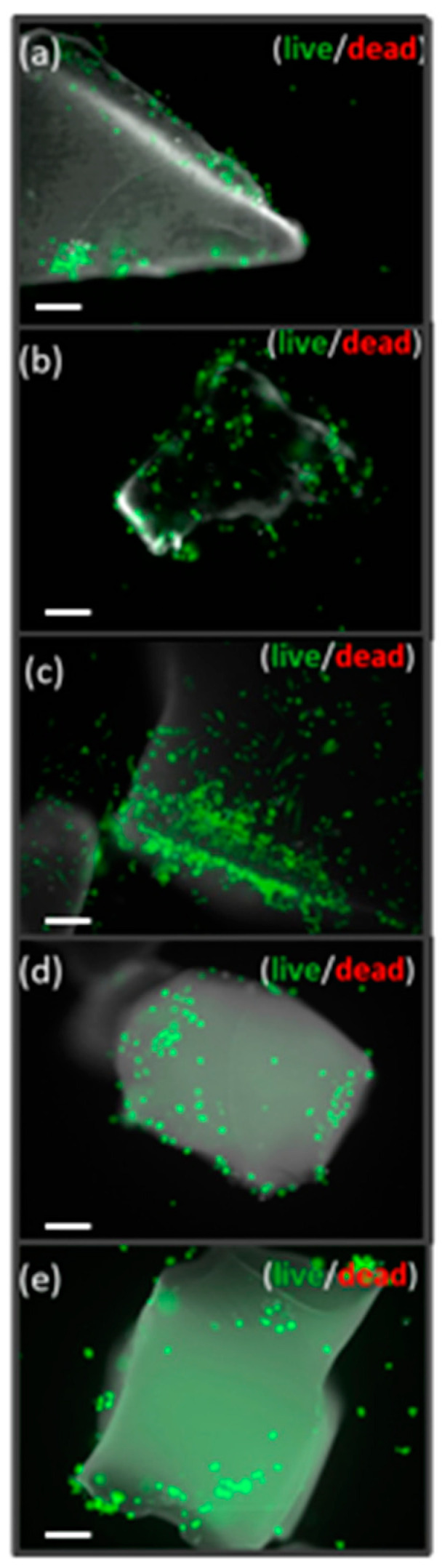
Live/dead staining of one-week HOB culture. HOB cultures with xerogels: SCS8 T10 (**a**), SCS8 T10 E1 (**b**), SCS8 T10 E7 (**c**), SCS8 E1 (**d**), and SCS8 E7 (**e**). Live cells appear green; the nuclei of dead cells stained in red and xerogels in gray. Scale bar represents 100 μm.

When HOB^®^ cells were seeded on the xerogels, an initial polarization could be seen from the first 24 h, followed by cell adhesion and morphological changes identified as initial markers of osteoblast differentiation and due to the presence of the biomaterial as described previously by us and others [44,53,75,76,77]. Adhesion, attachment, cell growth, and morphological changes in osteoblasts appeared to be substantially better in silica–chitosan xerogels’ experimental samples, mainly in those including TCP, than in cells grown on the bare substrata and revealed a successful cell attachment with marked morphological changes, such as filopodial and lamellipodial emission and an improved cell spreading. Cell viability at seeding is up to 98%. No significative apoptotic phenomena are detected either in the control or experimental groups. Live dead assay was performed to assess cell viability and growth and revealed that the majority of cells are in a viable state (green) at all time points, with only a few dead cells (red) (Figure 8). According to the data, SCS8T10_E7 xerogel presented the highest rate of proliferation and cell survival (see Table S3 and S4 and Figures S4 and S5 of the Supplementary Materials). In the presence of biomaterials, most cells migrate towards the xerogel and then adhere to the surface, while a lower percentage of cells adhere to the glass bottom of the well. As described below, cells grown in the presence of xerogels develop more efficient cell adhesion complexes as initial steps for differentiation, showing a time- and composition-dependent increase in number and size of focal adhesions. In control groups, cells expand and proliferate without significant migration anywhere (Figure 8).

### 2.8. Cell Morphology, Cytoskeletal

#### Organization and Focal Adhesions

**Figure 9:**
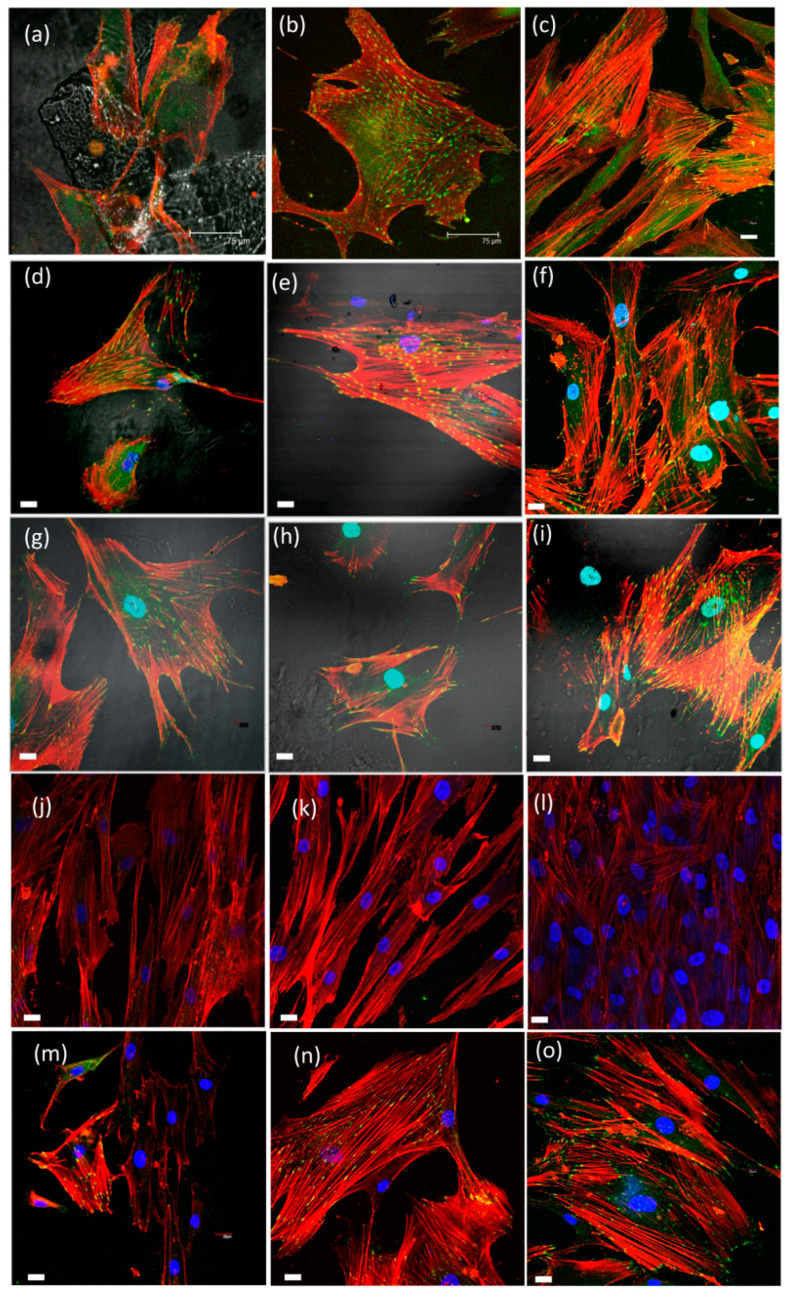
Representative images of HOB^®^ osteoblasts growing in the presence of: (**a**) xerogel SCS8T10_U, after 48 h in culture; (**b**) after 72 h in culture; (**c**) after 1 week in culture; (**d**) xerogel SCS8T10_E1, after 48 h in culture; (**e**) after 72 h in culture; (**f**) after 1 week in culture; (**g**) SCS8T10_E7, after 48 h in culture; (**h**) after 72 h in culture; (**i**) after 1 week in culture; (**j**) SCS8T20U after 48 h in culture; (**k**) after 72 h in culture; (**l**) after 1 week in culture; (**m**) control 48 h; (**n**) control 72 h; (**o**) control 1 week. Images acquired in the confocal microscope after immunolabelling of actin cytoskeleton with rhodamine phalloidin (fluoresces red) and vinculin (fluoresces green) for focal adhesions. Blue, DAPI-labelled nuclei. Unless specified, scale bar equals 20 μm.

**Figure 10:**
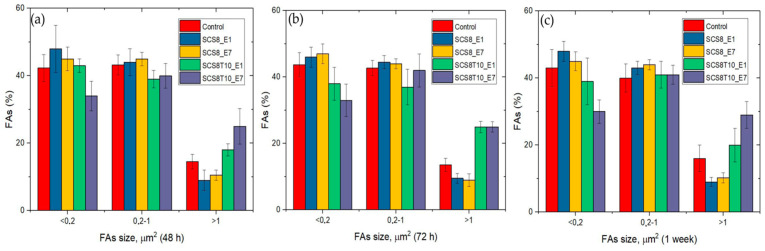
Time-dependent percentage of FAs according to size imaged in HOB^®^ cells grown on the described xerogels after (**a**) 48 h in culture, (**b**) 72 h in culture, (**c**) 1 week in culture. One-way analysis of variance. Statistical significance was defined as *p* < 0.05.

Spatiotemporal regulation of tension sustained at FAs has been described as essential for the regulation of cell migration and settlement pointing to extracellular matrix remodeling and new bone formation. Force-mediated FA signaling together with actin bundles organization in stress fibers regulates cell proliferation and differentiation [78,79,80,81,82,83]. Actin cytoskeleton immunolabelling in HOB^®^, after 48 h in culture in the presence of SCS8T10_U, revealed that the osteoblasts were big, some of them elongated, with filopodial and lamellipodial emissions. Some stress fibers arise into a well-developed actin cytoskeleton, and FA appears to be widely distributed, predominantly small, and on the tips of stress fibers. After 72 h, the number and size of focal adhesions and stress fibers increased, and also, the location changed, as focal adhesion tended to localize on the tips of stress fibers. A progressive approach of osteoblasts to biomaterial is observed with time, while the xerogel surface is covered and cells contact to neighbors as well as to material surface. After 1 week, mature focal adhesions, well-developed stress fibers, and cell-to-cell contacts become evident (see Figure 9a–c and Figure 10).

In HOB^®^/SCh8T10 E1 constructs, HOB^®^ cells elongated and developed numerous small- and medium-sized FAs, widely distributed, after 48 h in culture. After 72 h, cells spread with initial grouping and lamellipodial emissions. Additionally, stress fibers started to develop together with FAs, arranged on the tips of actin filaments, now reinforced as stress fibers. While in cells grown on SCS8T10_U, cell-to-cell contacts appear to be evident after 1 week, in SCS8T10_E1 constructs, cells started to group after 72 h, with notable maturation of focal adhesion and stress fibers. After 1 week, cells formed a complex reticular pattern with apparently more cells and were also more compact but less expanded than in previous days, with an increased number of medium-sized and mature FAs distributed along the tips of thicker and more evident stress fibers than in previous days and also than in control samples (see Figure 9d–f and Figure 10). When analyzing the variable area, perimeter, aspect ratio, circularity, and roundness of the selected ROIs, a highly significant difference (*p* < 0.05) appeared between cells grown on the different surfaces tested. Detailed data for morphological analysis are included in Figure S6, Tables S5 and S6 in the Supplementary Materials).

Cell assays after 48 h in culture in the presence of SCh8T10 E7 revealed the presence of elongated and longer cells quite more irregular in size than in SCh8T10E1 and control groups. Osteoblasts distributed in a reticular pattern, with lamellipodial and filopodial emissions trying to establish contact between neighboring cells. Widely distributed focal adhesions, although mainly peripherical, and also associated to the leading edge, as a marker of cell migration, were observed. In this group, mature focal adhesions sized >1 μm appeared in the first 48 h, increasing in number in a time-dependent way (Figure 9g–i). This distribution and also the size evolution during the time has been identified with cell migration [77,78,84].

Mostly elongated cells were also found after 72 h in culture, with less lamellipodial and more filopodial emissions than in previous days, oriented to material pieces. FAs are abundant—whether they are small-, medium-, and big-sized—and are occasionally distributed along the cell but mostly located in the cell periphery, associated to the tips of nascent stress fibers (Figure 9h,i). Control cells overlap in an intricated network with few focal adhesions mainly of small size. After 1 week, osteoblasts spread and increased in size, while establishing abundant contacts with neighboring cells (Figure S6, Tables S5 and S6 in the Supplementary Materials). FAs are numerous and big in size, mostly associated to stress fibers, thicker than in previous days. Mature and medium-sized FAs predominate in this group, and FAs sized >1 μm reached the highest value observed during the time in the tested xerogels. In control cells, actin cytoskeleton and occasional and small focal adhesions spread along the cell (see Figure 9 and Figure 10). The binding of vinculin to actin cytoskeleton is critical to its role in cell–matrix adhesion, and the overexpression of vinculin, forming big focal adhesions, is assumed as a marker of cell-to-substrata optimal adhesion [76,78,82,83,85].

HOB^®^ cells grown on SCS8T20_U appeared to be elongated after 48 h in culture, with scarce or no filopodial emission. Actin cytoskeleton becomes evident with initial stress fiber formation while immature punctate focal adhesions appear on tips of actin fibers. After 72 h in culture, the elongated cells display a reticular pattern, accentuated after 1 week with an increased number of elongated osteoblasts, tightly joined in a reticular pattern. No apparent filopodial or lamellipodial emissions allow a continuous sheet of osteoblasts in which some punctate or medium-sized FA can be observed on actin filament tips (Figure 9j–l).

**Figure 11:**
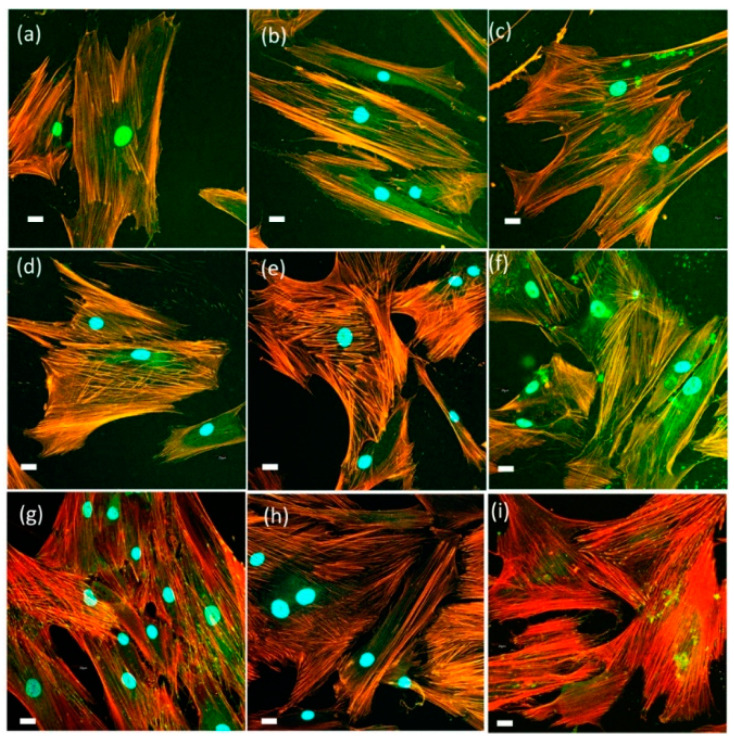
HOB^®^ human osteoblast in culture grown in the presence of xerogel SCS8_E1: (**a**) after 48 h, (**b**) after 72 h, and (**c**) after 1 week in culture. In (**d**–**f**) HOB^®^ grown in the presence of xerogel SCS8_E1 (**d**) after 48 h, (**e**) 72 h; (**f**) 1 week. In (**g**–**i**) control cells: after (**g**) 48 h, (**h**) 72 h; (**i**) 1 week. Images acquired in the confocal microscope after immunolabelling of actin cytoskeleton with rhodamine phalloidin (fluoresces red) and vinculin (fluoresces green) for focal adhesions. Blue, DAPI-labelled nuclei. Scale bar equals 20 μm.

HOB^®^ cells grown in the presence of SCS8_E1 and E7 appeared to be the widest in size at any experimental time. At 48 or 72 h in culture, cells did not show evident filopodial emissions, focal adhesion of small- and medium-sized predominates and stress fiber development is scarce, while cell-to-cell contact appeared earlier than in the SCS8T10 or SCS8T20 groups. After 1 week, no mature focal adhesions appear in a significant way (Figure 10). Actin cytoskeleton organize mostly in periphery, with a few stress fibers tipped with medium-sized focal adhesions (Figure 11). In a similar way, cells grown on SCS8_E7 are wide in size and contact each other from 48 h onwards forming a complex reticular pattern. Stress fiber development is more evident than in the SCS8 E1, but to a lesser extent than in other experimental groups and, also, distribution differs, located in the cell center, not associated to cell prolongations or mature focal adhesions. From 72 h onwards, the actin stress fiber pattern turns to periphery, and after 1 week, cells appeared to cover the surface but without the significant presence of mature focal adhesions (Figure 10 and Figure 11).

The comparative analysis of FA maturation based on FA size revealed a highlighted role of TCP, with significant differences (*p* < 0.05) between experimental and control groups, and also between silica/CS/TCP hybrid xerogels and those without TCP. Data confirmed qualitative observations (Figure 8, Figure 9 and Figure 11) and revealed that control cells and cells grown on SCS8_E1/E7 developed a higher percentage of punctate, non-mature, i.e., <0.2 μm^2^ FAs than cells grown on SCS8T10_E1/E7 after 48 h, 72 h, and one week. In contrast, the percentage of mature FAs sized >1 μm^2^ was significantly higher in the SCS8T10_E1 and E7 constructs than in groups without TCP, mainly after 72 h and one week of culture. In addition, the sample SCS8T10_E7 presents a higher percentage of FAs >1 μm^2^ than its equivalent with only 1 day of washing.

### 2.9. Mineralization

Alizarin red staining (ARS) is a well-established method to characterize a mineralized matrix due to the differentiation of osteogenic lineage cells [86,87]. As shown in Figure S7 in the Supplementary Materials, ARS staining allows the simultaneous evaluation of mineral distribution and inspection of structures by optical microscopy. Initially, HOB^®^ cells grown on SCS8 U were examined after 21 days, and scarce mineralization was found. SCS8T10 and SCS8T10 E7 samples were then selected for a deepest assay for one more week. After 28 days in culture, the staining of the constructs with ARS revealed a deeply stained mineralized layer with calcium deposits within cells and the extracellular matrix, clearly visualized under 10× magnification. Ongoing studies in our lab are focused on analyzing the samples during a longer period.

Bone tissue has a unique capacity to regenerate in response to injuries in a dynamic process in which osteoblasts play a principal role. During osteogenesis and regeneration processes, osteoblasts differentiate into osteocytes, post-mitotic cells of the osteoblast lineage capable of synthesizing both the osteoid and fibers, deeply embedded within extracellular bone matrix. Osteocytes represent the final differentiation step in the process: osteoblasts lose a large part of their cell organelles but, in turn, gain long, thin, and branched cell processes by which the cells remain in contact with neighboring osteocytes, capillaries, and lining osteoblasts. Osteocytes represent more than 90% of the bone cell in the adult skeleton, they have a long life, up to 25 years, while osteoblasts have a mean life of 150 days and become the primary mechano-sensors in bone [88,89]. One of the key pieces in the mechano-transduction process appears to be the so-called focal adhesions, as we have previously demonstrated with silica–chitosan aerogels. Focal adhesions (FAs) are complex intracellular linkages between integrins and the F-actin cytoskeleton that both transmit and respond to mechanical forces. FAs show complex mechano-sensitivity such that they form or enlarge when force increases, and shrink or disassemble when force decreases. In this landscape, vinculin seems to be a key element in the molecular “clutch” that links the actin cytoskeleton and extracellular matrix and co-localizes with areas of high force during leading edge protrusion and have also been involved in regulatory mechanisms in which the ability of vinculin to bear force determines whether adhesions assemble or disassemble under tension [90]. Both osteoblast behavior and cytoskeletal arrangement including vinculin expression in response to preselected xerogels revealed a positive effect of the proposed biomaterials as scaffolds for bone tissue regeneration, as detailed.

The composition of silica xerogels can be tuned so that they elicit bone bioactive characteristics [44,53]. As it is widely accepted, the bone-bonding ability of a biomaterial depends on ion release and exchange to develop a biological apatite layer when these materials are in the presence of body fluids. As a natural polymer, chitosan offers a unique set of characteristics for developing advanced functions such as biocompatibility, biodegradability, hydrophilicity, and nontoxicity. Phosphorylation of chitosan nanofibrous scaffolds has been shown to improve proliferation and osteogenic differentiation of human osteoblast-like cells. Phosphorous incorporation into polymer scaffolds has been shown to improve interaction with a wide variety of cell types as well as to influence their attachment and function both in vitro and in vivo. In particular, the ability of phosphorous groups to bind calcium and proteins, their positive influence on osteoblastic cells, and their wide range of mechanical properties make phosphorous-containing polymers promising materials for bone regeneration [24,25].

An ideal bone scaffold for clinical use should be made of biomaterials that imitate the structure and properties of natural bone extracellular matrix and provide at least most of the necessary environmental cues found in natural bone. Furthermore, sterilization capability should be achieved without substantial physicochemical or structural damage to the scaffold. Taking these premises into account, our findings reveal that ethanol-washed samples showed excellent bioactivity and biological response as a consequence of the synergistic effects of washing process, large surface area, and porous structure. Additionally, the positive role of calcium and phosphate inducing osteoblast growth and differentiation into an osteocyte-like morphology [89] was confirmed for the results obtained in TCP containing samples [5,24].

On the other side, the pore structure of water-washed (W30) samples partially collapsed during evaporative drying, provoking a decrease in the pore size towards the micropore domain. Nevertheless, a good bioactive response was still present in all cases. However, how this combination of processing and structural features affects the biological behaviors of these silica/chitosan/TCP xerogels, and the way each parameter contributes, is still unclear and will be the focus of future investigation. The results presented here implicate that an ethanol-washed scaffold built of mesoporous silica/chitosan/TCP has an attractive design for bone tissue engineering, appearing to be responsible for cell changes compatible with osteoblast differentiation and are promising candidates for clinical use, although a number of steps are still to be overcome.

## 3. Materials and Methods

### 3.1. Materials

Chitosan (CS; 50,000–190,000 Da; 75–85% deacetylation degree) was supplied by Sigma Aldrich (St. Louis, MI, USA). Tetraethylortosilicate (TEOS, 99%) and hydrochloric acid (37%, Pharma grade) were purchased from Alfa Aesar (Haverhill, MA, USA). Tri-calciumphosphate (TCP, pure, pharma grade) and absolute ethanol (99.5%) were obtained from Panreac (Barcelona, Spain), HOB^®^ human osteoblasts, fetal calf serum, and Osteoblast Growing Medium (Promocell, Heidelberg, Germany) Paraformaldehyde, PBS, Triton x-100, bovine serum albumin, Metanol, rhodamine phalloidin, and monoclonal anti-vinculin FITC conjugate were all purchased from Sigma Aldrich, (St. Louis, MI, USA) and Hard Set Vectashield with DAPI^®^ (Vector, Burlingame, CA, USA).

### 3.2. Synthesis of SiO_2_/CS and SiO_2_/CS/TCP Xerogels

SiO_2_/CS xerogels were synthesized by a sol-gel route to produce inorganic/organic hybrid sols covering a range of chitosan nominal content up to 20 *w*%. TEOS and CS biopolymer were used as gel precursors, and the preparation procedure is as follows: firstly, a silica sol was prepared by mixing TEOS/water/HCl in a molar ratio of 1:4:0.05. The mixture was hydrolyzed under the catalyst effects of ultrasounds in a glass reactor by supplying 4.5 kJ/cm^3^ of ultrasonic energy density, using a Vibracell 600 Watt ultrasonic processor from Sonics and Materials (Newton, CT, USA). A second sol was obtained by dissolving CS powder in 0.6 M HCl by exposure to 10 kJ/cm^3^ of ultrasound energy, to produce 2% *w*/*v* CS solution. Next, different amounts of the CS sol were mixed with the hydrolyzed TEOS, under 0.5 kJ/cm^3^ additional dose of ultrasound energy, resulting in clear homogeneous solutions with different silica/CS weight ratios, named SCSx (x = 0, 4, 8, 12, and 20 of CS *w*%). At this moment, 0.6 M HCl was added to the hydrolyzed TEOS/CS mixture, so that the molar ratio of water/TEOS was kept constant at 30:1 in all samples.

Additionally, to study the effect of calcium and phosphorous on the biomineralization of the samples, different amounts of TCP were added to a hybrid sol of pre-determined fix composition (SCS8), thus producing SCS8Ty Ca–P-containing samples, with y = 10, 20 TCP wt.%. Then, the liquid homogeneous sols were poured into cylindrical vials, hermetically closed, and placed in an oven at a controlled temperature of 50 °C, where the gelation process occurred within 24–48 h of heating. Next, the resulting gels were aged in its mother liquid at 50 °C for 14 days to strengthen.

Finally, the aged gels were washed in order to remove traces of HCl or unreacted precursors according to three different processes: by soaking in absolute ethanol at 50 °C for one day (E1 samples) or for 7 days (E7 samples) and, finally, using distilled water as a washing solvent for 30 days (W30). Next, the samples were dried by evaporation for 48 h at 80 °C in a controlled-temperature oven at ambient air pressure.

**Figure 12:**
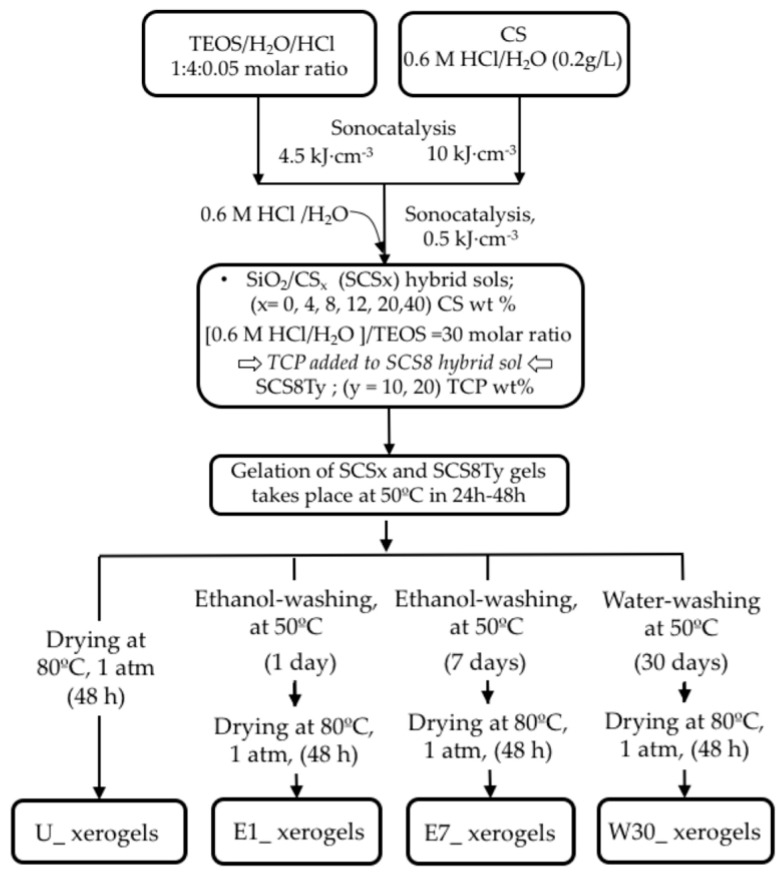
Scheme of the synthesis procedure for SiO_2_/CS/TCP xerogels following different washing treatments before evaporative drying.

According to these procedures, different xerogel sample series, associated to the three different washing procedures performed, were obtained, e.g., SCSx_E1, SCSx_E7, SCSx_W30, SCS8Ty_E1, SCS8Ty_E7, and SCS8Ty_W30. Besides, a fourth set of dried unwashed xerogels (SCSx_U) samples was included in the study, just for comparison. Monolithic cylinder samples with 3–5 mm diameter and 5–8 mm height were obtained after evaporative drying at 80 °C and ambient pressure (Figure S1 of the Supplementary Materials). A flow chart of the synthesis in Figure 12 gives a global view of the entire fabrication process of xerogels.

To sum up, we synthesized different xerogels with varied inorganic (SiO_2_)/organic (chitosan) and TCP weight ratios and classified them in four sample series, according to the washing processing before evaporative drying: U, E1, E7, and W30.

### 3.3. Materials Characterization

Several structural, textural, and morphological techniques were used to characterize the xerogels, obtained as monolithic cylinder samples. The bulk density was determined from geometrical measurements, calculating the mass and size of cylindrical samples with a microbalance (precision ± 0.1 mg) and a slide caliper (±0.05 mm accuracy).

The surface chemical structure was studied by Fourier transform infrared spectroscopy (FTIR) using an IR Bruker Tensor 37 spectrometer (Billerica, MA, USA), and the transmission was measured at wavelengths ranging from 4000 to 400 cm^−1^, with a resolution of 4 cm^−1^ and an average of 100 scans. The samples were stored overnight in a stove at 60 °C, then ground and mixed with KBr, and pressed into a self-supporting wafer. The wafer was put on a sample holder for spectrum collection.

Thermogravimetric analysis (TGA) was performed with a TGA Q50 from TA instruments (New Castle, DE, USA). A temperature ramp of 50 to 900 °C under air atmosphere was used with a sample heating rate of 10 °C·min^−1^.

Determination of carbon and nitrogen was made using an Euro 3024 EA Elemental Analyzer (EuroVector), with a 0.5 μg threshold for each element. The resulting EA nitrogen values must provide information about the hybridization reaction efficiency between the organic (CS) and inorganic (TEOS) precursor components, as CS is the unique source of nitrogen in the samples.

Nitrogen physisorption experiments were conducted at 77 K using a Micromeritics ASAP 2010 analyzer (Norcross, GA, USA), equipped with a pressure transducer resolution of 10^−4^ mm Hg. Adsorption–desorption isotherms of N_2_ were recorded after grinding and degassing the samples at 120 °C for 6 h. The Brunauer–Emmett–Teller (BET) theory was used to calculate specific surfaces areas of the xerogels in the range 0.05 < P/P_0_ < 0.3 and BET C-constants were calculated according to the multi-point BET method [91]. The pore size and pore volume were estimated from the nitrogen desorption isotherms, based on the BJH model [92].

Micropore volumes were determined on the basis of the t-plot method (de Boer) to nitrogen adsorption data. The isotherms of xerogel samples were transformed into t-plots [93] by converting their relative pressures into t-values, according to Harkins–Jura equation [57,61,62]:

t = (0.1) × [13.99/(log (P_0_/P) + 0.034)]^1/2^ (3)

where t (in nm) describes the statistical thickness of the adsorbed nitrogen film on a flat surface of a particular nonporous adsorbent, which is taken as a standard reference, while 13.99 and 0.034 are experimental parameters for alumina-based non-porous reference solids (standard isotherm) [94].

### 3.4. In Vitro Degradation: Determination of Ca and Si Release and Measurement of pH

The quantification of the calcium and silicon release was performed using inductively coupled plasma (ICP) with a Thermo Scientific IRIS Intrepid II instrument. On purpose, xerogel powders were immersed in phosphate buffered saline (PBS), a buffer solution (constant pH = 7.4) frequently used in biological research, since the osmolarity and ion concentrations of the solution usually match those of the human body. PBS tablets from Sigma were dissolved in 200 mL of ultrapure water MiliQ, at 37 °C for 1 week, with a concentration of 1 mg/mL and without soaking refresh. Plastic vials of 6 mL capacity were used, and 4 mL aliquots of PBS incubated solution were removed at predetermined points of 12 h, 1, 2, 3, 5, and 7 days. For this process, a 0.45 μm membrane filter Millipore was used, and the aliquots were placed in clean plastic vials to avoid any type of contamination and stored at 4 °C.

The pH was determined at the same time points by placing a clean and calibrated pH meter probe (HACH sensIONTM + pH = 3, with resolution pH of 0.01) into a PBS standard solution containing a sample concentration of 2 mg/mL, stored at 37 °C, then recording the stabilized value. Ca and Si released concentrations, and the pH of samples was calculated using the average of all triplicates of the samples.

### 3.5. In Vitro Biomineralization in SBF

An in vitro biomineralization study was conducted to examine the ability of the xerogels to form hydroxyapatite. The procedure followed the method reported by Kokubo et al. [95], using a simulated body fluid (SBF) where samples were immersed in polyethylene vials for various soaking times: 7, 14, 21, and 28 days. The test was performed with fluid weekly exchange, and the samples were washed with distilled water after soaking and then drying in an oven at 80 °C overnight for further characterization.

The biomineralization was evaluated by confirming the formation of an apatite-like layer on the surface of the xerogels after immersion. To this end, a field-emission scanning electron microscopy (FEI Nova NanoSEM 450; resolution 1.4 nm), fitted with a Bruker SDD-EDS detector for determining compositional differences across the specimen surface, was used. The samples were mounted on standard aluminum stubs, fixed with conductive double sided carbon adhesive tape. To reduce charging effects, a 10 nm gold coating layer was deposited on the surface for all samples.

### 3.6. Cell Culture

HOB^®^ cells were seeded on the preselected scaffolds under sterile conditions. Once reached, the optimal confluence was detached, counted to optimal cell density, and analyzed for cell viability (automated Luna^®^ cell counter, Invitrogen). Cells did not exceed ten population doublings. In order to achieve optimal sterilization, xerogels were sterilized in a clinically standardized autoclave prior to cell seeding, in order to achieve optimal sterilization, according to European standard DIN EN ISO 13060 recommendations for class B autoclaves. Once sterilized, the samples were placed under sterile conditions in a laminar flow chamber on Mattek^®^ glass-bottom wells. A drop of 50 μL of cell suspension at a density of 15,000 HOB^®^ cells/cm^2^ was added to each sample and kept for 30 min under incubation in humid conditions at 37 °C and 5% CO_2_ to ensure optimal cell attachment and avoid dispersion. Wells were then filled with OGM^®^ supplemented to a final concentration of 0.1 mL/mL of fetal calf serum at 37 °C and 5% CO_2_ and incubated during experimental times. The growth medium was changed every two days and collected for the determination of degradation products. The test groups were as follows: SCS8, SCS8T10, SCS8T20, and controls grown on glass. HOB^®^ cells grown on glass were used as a control.

### 3.7. Live/Dead Cell Assay

Live/dead cell assay was performed to evaluate the viability/cytotoxicity of HOB cells grown on the silica/chitosan xerogels. After being incubated for 24 h, 48 h, and 7 days, the cell/scaffold constructs were rinsed with PBS twice and then stained with calcein-AM (0.5 μL/mL) in PBS and ethidium homodimer-1 (EthD-1) (2 μL/mL) in PBS to display the live and dead cells, respectively. The cell/scaffold constructs were observed on the confocal laser scanning microscope (CSLM).

### 3.8. Cell Morphology and Spreading

Cells were daily examined with the phase-contrast microscope in order to evaluate cell morphology, alignment distribution, and spreading. The initial adhesion phase to surfaces was assessed prior to immunolabeling for fluorescence and confocal laser scanning microscope examination of the experimental and control samples. Both fluorescence and confocal examination combined, when possible, with fluorescence and Nomarski modes in order to acquire both material and growing cells.

### 3.9. Actin Cytoskeletal Organization

After being incubated for 24 h, 48 h, and 7 days, osteoblasts were immunolabeled after 24 h, 48 h, and 1 week with rhodamine–phalloidin and vinculin in order to assess cytoskeletal changes and focal adhesion development. Cells were washed with prewarmed phosphate buffered saline (PBS), pH = 7.4, and fixed with 3.7% paraformaldehyde at room temperature, washed, and then, permeabilized with 0.1% Triton X100). After washing, cells were preincubated with 1% bovine serum albumin (Sigma) in PBS for 20 min prior to cell immunolabelling for actin cytoskeleton with rhodamine phalloidin (Sigma). After 20 min, samples were rinsed with prewarmed PBS prior to mounting with Vectashield^®^ (Vector, Burlingame, CA, USA). At least, five samples of each type were seeded and analyzed per experiment. The test groups were as follows: SCS8, SCS8T10, SCS8T20, and control. HOB^®^ cells grown on glass under conditions described above were used as control. The measurement of focal adhesion size and location was conducted for at least 10 cells on each substrate.

### 3.10. Confocal Examination

Samples were visualized using an Olympus confocal microscope. At least five samples were analyzed for each group to assess surface influence on cytoskeletal organization, focal adhesion number, and development and cell morphology. Images were collected and processed using imaging software. At least 50 cells per sample were analyzed. Samples were exposed to the lowest laser power that was able to produce a fluorescent signal for a time interval not higher than 5 min to avoid photobleaching. A pinhole of 1 Airy unit was used. Images were acquired at a resolution of 1024 × 1024.

### 3.11. Image Analysis

To analyze the differences in focal adhesion number between different sample groups, images were collected and processed. Area, perimeter, roundness, circularity, and aspect ratio were analyzed as shape variables. Sample images were collected as frames obtained at 40× magnification and processed using Image J software (NIH, http://rsb.info.nih.gov/ij (accessed on 28 July 2021)). For quantitative analysis, there were at least 40 regions of interest (ROIs). All of the ROIs are cells selected under the following criteria: well-defined limits, clear identification of nucleus, and absence of intersection with neighboring cells. All experiments were repeated in triplicates unless otherwise stated. All data were SPSS analyzed and expressed as the mean ± standard deviation. Once normality and homoscedasticity were confirmed, the difference between the mean values was analyzed under a one-way analysis of variance, Brown–Forsythe, and Games–Howell tests. Statistical significance was defined as *p* < 0.05.

### 3.12. Mineralization

Mineralization was induced on xerogel–HOB^®^ constructs by the addition of Osteoblast Mineralization Medium (Promocell, Heidelberg, Germany) for at least 21 days. HOB^®^ cultured with Osteoblast Growth Medium (Promocell, Heidelberg, Germany) was used as negative control, and cells grown with Osteoblast Mineralization Medium (Promocell, Heidelberg, Germany) were used as positive mineralization control. Media were changed three times per week, and cell cultures were incubated at 37 °C and 5% CO_2_.

### 3.13. Detection of Mineralization

Mineralization capability was analyzed after 28 days under culture by Alizarin Red Staining Solution (Fisher Scientific, Waltham, MA, USA). Briefly, all cells were fixed with 4% paraformaldehyde for 1 h at room temperature, washed 3 times in phosphate-buffered saline, stained with 2% alizarin red solution, and again, rinsed 3 times in phosphate-buffered saline. Calcium deposits within cells and extracellular matrix were visualized under fluorescence microscope (magnification 10×). All experiments were performed in triplicate.

## 4. Conclusions

SiO_2_/CS and SiO_2_/CS/TCP micro- and mesoporous hybrid xerogels with high surface areas about 800 m^2^·g^−1^ and interconnected porosity were obtained by the sol-gel synthesis introduced in this work. Using ethanol as a washing solvent resulted in an increase in surface area, pore volume, and pore size, compared to a higher soaking time water-washing procedure, which induced some structural changes mediated by surface silanols and created 5–25% microporosity in the xerogels. Compared with water-washed xerogels, ethanol-washed xerogels underwent biodegradation and the release of both Ca and Si ions to the free medium. However, in the case of water-washed samples, only the release of Si ions was observed. Furthermore, the ability to induce and control the growth of a bioactive layer formed by HAp spherulites from submicrometric to 10 μm in diameter after 28 days of soaking in SBF was improved for CaPs-containing samples and served to promote the adhesion and proliferation of osteoblasts, contributing efficiently in the bone repairing process. Further understanding of this novel bioactive system as a platform for attachment, spreading, and proliferating cells will be important for the administration of silica hybrid CaPs-based implants to improve bone tissue engineering.

## Figures and Tables

**Table 1:** C and N results obtained from elemental analysis of SiO_2_/CS/TCP xerogels (SCSx and SCSxTy sample series), according to the washing procedure performed before evaporative drying (U, E1, E7, or W); also, the C/N ratio and CS real content, estimated from N values obtained from EA, are listed; x and y in sample code refers to the nominal content of CS and TCP added amount, respectively; (x = 0, 4, 8, 12, 16, 20, and 40 CS wt.%; y = 10, 20 TCP wt.%).

Xerogel Samples	Unwashed (U)				Ethanol-Washed_1 Day (E1)				Ethanol-Washed_7 Days (E7)				Water-Washed_30 Days (W30)	
	C (wt%)	N (wt%)	C/N	Chitosan (wt%)	C (wt%)	N (wt%)	C/N	Chitosan (wt%)	C (wt%)	N (wt%)	C/N	Chitosan (wt%)	C (wt%)	N (wt%)
Pure SiO_2_	0.74	---	---	---	---	---	---	---	---	---	---	---	0.07	
SCS4	1.92	---	---	---	4.11	0.07	58.7	0.89	3.71	---	---	---	0.35	---
SCS8	3.11	0.12	25.9	1.53	4.46	0.21	21.2	2.67	4.73	0.05	89.2	0.67	0.17	---
SCS12	4.55	0.38	12.0	2.44	4.60	0.27	17.0	3.43	--	--	--	--	0.31	---
SCS16	5.35	0.54	9.9	6.87	4.85	0.32	15.1	4.08	5.73	0.24	23.9	3.05	0.40	---
SCS20	4.98	0.45	11.1	5.73	5.80	0.48	12.1	6.11	5.41	0.19	28.5	2.40	0.34	---
SCS40	8.81	1.21	7.6	15.40	--	--	--	--	--	--	--	--	0.80	---
SCS8T10	2.71	0.13	20.8	1.65	4.02	0.25	16.1	3.18	4.25	0.12	35.4	1.50	0.12	---
SCS8T20	2.44	0.08	24.4	1.27	4.38	0.23	19.0	2.93	4.47	0.12	37.2	1.52	0.10	---

The real CS content was calculated from the N value measurements in EA, considering that chitosan is the unique source of nitrogen in the samples. Standard deviation was less than 0.3%.

**Table 2:** Bulk density and textural data of SiO_2_/CS/TCP xerogels subjected to different washing treatments (U, E1, E7, and W30) before drying.

Sample	Unwashed				Ethanol-Washed								Water-Washed			
	(U)				1 Day Ethanol-Soaking (E1)				7 Days Ethanol-Soaking (E7)				30 Days Water-Soaking (W30)			
	ρ (g/cm^3^)	S_BET_ (m^2^/g)	V_p_ (cm^3^/g)	D (nm)	ρ (g/cm^3^)	S_BET_ (m^2^/g)	V_p_ (cm^3^/g)	D (nm)	ρ (g/cm^3^)	S_BET_ (m^2^/g)	V_p_ (cm^3^/g)	D (nm)	ρ (g/cm^3^)	S_BET_ (m^2^/g)	V_p_ (cm^3^/g)	D (nm)
**SCS8**	1.04 ± 0.03	653.0	0.58	3.3	0.36 ± 0.05	826.7	1.47	6.7	0.24 ± 0.05	815.1	1.64	7.1	1.38 ± 0.05	741.0	0.40	2.5
**SCS8T10**	1.20 ± 0.03	485.5	0.43	3.3	0.58 ± 0.01	821.2	1.23	5.5	0.53 ± 0.02	870.0	1.49	6.3	1.43 ± 0.01	662.2	0.39	2.7
**SCS8T20**	1.53 ± 0.03	360.8	0.28	3.0	0.63 ± 0.02	728.4	0.91	4.7	0.56 ± 0.01	887.6	1.40	6.0	1.48 ± 0.03	663.7	0.42	2.7

Correlation coefficient for BET surface area measurements was higher than 0.9995 in all cases.

**Table 3:** t-plot parameters of SCS8, SCS8T10, and SCS8T20_W30 xerogels.

Sample	BET			t-Plot						r_1_/r_2_
	S_BET_ (m^2^/g)	V_p_ (g/cm^−3^)	D (nm)	S_tot_ (m^2^/g)	S_meso_ (m^2^/g)	S_mic_ (m^2^/g)	S_Ext_ (m^2^/g)	V_Tot_ (cm^3^/g)	V_micro_ (cm^3^/g)	r_1_/r_2_
**SCS8_W30**	741.0	0.40	2.5	537.0	531.6	204.0	5.4	0.39	0.10	0.9971/0.9929
**SCS8T10_W30**	662.2	0.39	2.7	529.8	525.2	132.4	4.6	0.38	0.06	0.9989/0.9913
**SCS8T20_W30**	663.7	0.42	2.7	623.8	620.9	39.9	2.9	0.41	0.02	0.9997/0.9919

r_1_ and r_2_ are the regression coefficients of linear fitting for the first (0.35 nm < t < 0.5 nm) and second linear regime (t > 1.0 nm) of the t-plot, respectively.

**Table 4:** Surface composition by EDS analysis of the various xerogels used in this study.

	Sample	Ca (at %)	P (at %)	O (at %)	Ca/P Ratio
As prepared	SCS8T10_(w)	--	--	35.98	--
	SCS8T10_(u)	3.57	2.60	67.12	1.37
4 weeks soaking in SBF	SCS8_(w)	6.48	3.44	22.90	1.88
	SCS8T20_(w)	5.20	3.64	45.82	1.43
	SCS8T10_(e1d)	8.52	4.61	42.21	1.85
	SCS8T20_(e1d)	9.23	5.85	38.48	1.58
	SCS8T10_(e7d)	11.45	8.38	46.59	1.37
	SCS8T20_(e7d)	17.60	7.80	42.08	2.25

Standard deviation less than 1%.

## Supplementary Materials

The following are available online at https://www.mdpi.com/article/10.3390/ijms22158321/s1, Figure S1: Images of SiO_2_/CS and SiO_2_/CS/TCP hybrid xerogels obtained by evaporative drying at 80 °C and ambient air pressure, Figure S2: pH time evolution of xerogels soaked in PBS, Figure S3: Elemental mapping at the microstructural level by scanning electron microscopy (SEM) with energy dispersive X-ray spectrometry, Figure S4: Live/dead assay. Positive and negative controls, SM 7, Figure S5: Live/dead assay. Column graphs, Figure S6: Shape variables, SM13, Figure S7: Mineralization of SCS8T10 E1 and SCS8T10_E7, Table S1: Bulk density and textural data from N_2_ physisorption of SiO_2_/CS and SiO_2_/CS/TCP hybrid xerogels, Table S2: BET and t-plot textural parameters for SCSxTy_W30 xerogels, Table S3: Quantitative data obtained after live/dead cell analysis. Live and dead data represent positive and negative controls, respectively, Table S4: Raw data from Table S3, Table S5: Significant differences between shape variables inter groups, Table S6: Pairwise differences between groups with 95% confidence interval.
