# Supplementary material for: Effect of Washing Treatment on the Textural Properties and Bioactivity of Silica/Chitosan/TCP Xerogels for Bone Regeneration
Source: Int J Mol Sci. 2021 Aug 2;22(15):8321. doi: 10.3390/ijms22158321 (PMC8347756; doi:10.3390/ijms22158321)
Supplement: Supplementary file 1 [file ijms-22-08321-s001.zip › ijms-1273170-supplementary.pdf]

Effect of washing treatment on the textural properties and bioactivity of silica/chitosan/TCP xerogels for bone regeneration

Antonio Pérez-Moreno, María Virtudes Reyes-Peces, José Ignacio Vilches-Pérez, Rafael Fernández-Montesinos, Gonzalo Pinaglia-Tobaruela, Mercedes Salido, Nicolás de la Rosa-Fox and Manuel Piñero

- **SUPPLEMENTARY MATERIAL**

SM1. Figure S1. Images of SiO_2/CS and SiO_2/CS/TCP hybrid xerogels obtained by evaporative drying at 80 ºC and ambient air pressure

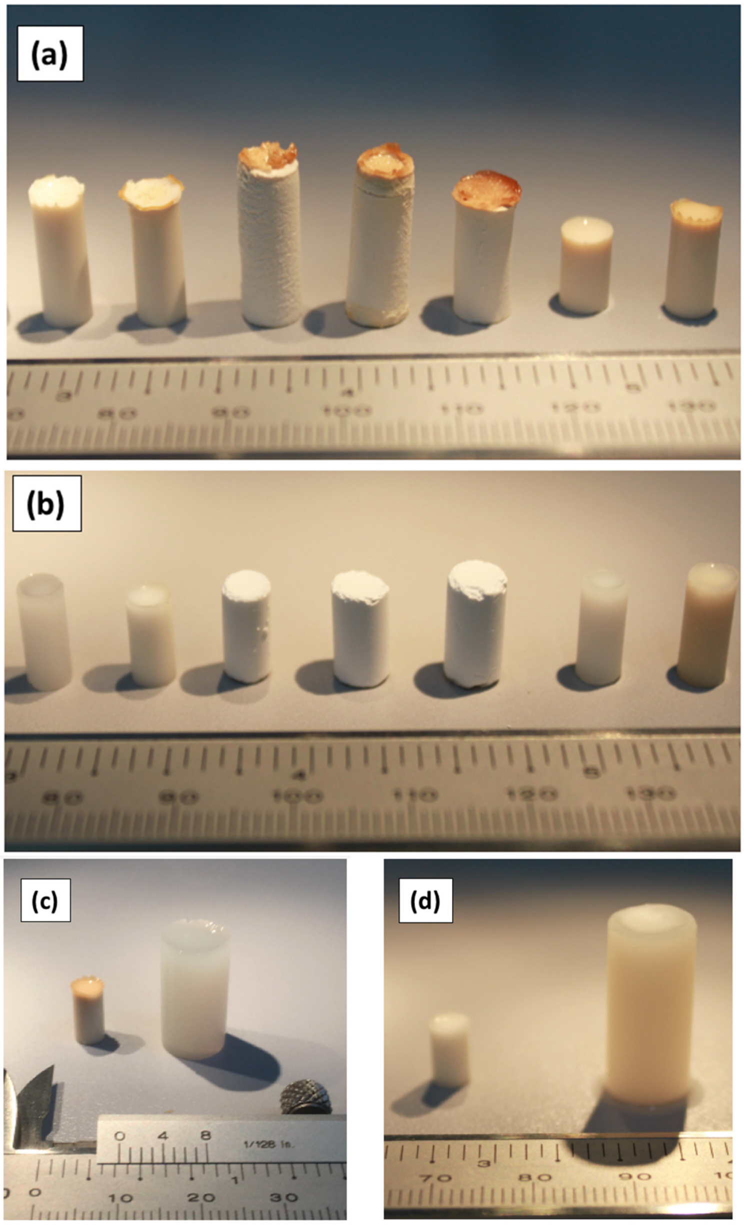

Figure. S1. Images of SCS4, SCS8, SCS16, SCS20, SCS40, SCS8T10 and SCS8T20 xerogels (a) Unwashed (U), and (b) Ethanol- washed for 7 days (E7); and images of gels with the same composition: in dry state (xerogel), at the left, and in wet gel state, at the right, for both: (c) SCS8T10_U; (d) SCS8_E7 specimens, showing the great shrinkage underwent during evaporative drying at 80 ºC

SM2. Table S1. Bulk density and textural data from N_2-physisorption of SiO_2/CS and SiO_2/CS/TCP hybrid xerogels

Table S1. Bulk density and BET textural parameters for SCSx and SCSxTy xerogels

Sample	Unwashed				Ethanol-washed								Water-washed			
					1 day ethanol-soaking E1				7 days ethanol-soaking E7				30 days water- soaking W30			
	ρ (g/cm^{-3})	S_{BET} (m^2/g)	Vp (cm^{-3}/g)	D (nm)	ρ (g/cm^{-3})	S_{BET} (m^2/g)	Vp (cm^{-3}/g)	D (nm)	ρ (g/cm^{-3})	S_{BET} (m^2/g)	Vp (cm^{-3}/g)	D (nm)	ρ (g/cm^{-3})	S_{BET} (m^2/g)	Vp (cm^{-3}/g)	D (nm)
Pure SiO_2 xerogel	0.79±0.01	668.5	0.81	4.5	---	---	---	---	---	---	---	--	1.22±0.02	827.4	0.47	2.6
SCS4	1.04±0.06	653.3	0.58	3.4	0.53±0.02	836.8	1.30	5.7	0.53±0.02	834.4	1.43	6.2	1.58±0.02	747.4	0.45	2.9
SCS8	1.04±0.03	653.0	0.58	3.3	0.36±0.05	826.7	1.47	6.7	0.24±0.05	815.1	1.64	7.1	1.38±0.05	741.0	0.40	2.5
SCS12	0.78±0.03	653.7	0.56	3.3	0.27±0.01	802.7	1.43	6.6	0.33±0.01	857.4	1.68	7.5	1.39±0.01	757.1	0.42	2.6
SCS20	0.71±0.04	592.0	0.48	3.2	0.28±0.03	852.4	1.31	5.7	0.25±0.03	867.6	1.70	7.2	1.33±0.03	737.2	0.40	2.9
SCS40	0.64±0.02	523.1	0.46	3.3	---	---	---	---	---	---	---	---	1.28±0.02	687.3	0.43	2.9
SCS8T10	1.20±0.03	485.5	0.43	3.3	0.58±0.01	821.2	1.23	5.5	0.53±0.02	870.0	1.49	6.3	1.43±0.01	662.2	0.39	2.7
SCS8T20	1.53±0.03	360.8	0.28	3.0	0.63±0.02	728.4	0.91	4.7	0.56±0.01	887.6	1.40	6.0	1.48±0.03	663.7	0.42	2.7

SM3. Table S2. BET and t-Plot textural parameters for SCSxTy_W30 xerogels.

Table S2. BET and t-plot textural parameters for W30 xerogels. (r_1 and r_2 are the coefficients from linear regression in t-plot for the first (0.35 nm < t < 0.5 nm) and second linear regime (t > 1.0 nm) , respectively)

Sample	BET			t-plot						r_1 / r_2
	S_{BET} (m^2/g)	V_p (g/cm^{-3})	D (nm)	S_{tot} (m^2/g)	S_{meso} (m^2/g)	S_{mic} (m^2/g)	S_{Ext} (m^2/g)	V_{Tot} (cm^3/g)	V_{micro} (cm^3/g)	r_1 / r_2
SCh0_W30	827.4	0.47	2.6	691.3	686.5	136.1	4.8	0.47	0.06	0.9987/0.9899
SCh4_W30	747.4	0.45	2.9	679.6	672.8	67.8	6.8	0.44	0.03	0.9996/0.9859
SCh8_W30	741.0	0.40	2.5	537.0	531.6	204.0	5.4	0.39	0.10	0.9971/0.9929
SCh12_W30	757.1	0.42	2.6	571.0	564.7	186.1	6.3	0.40	0.09	0.9974/0.9988
SCh20_W30	737.2	0.40	2.9	558.4	551.6	178.8	6.8	0.39	0.09	0.9973/0.9994
SCh40_W30	687.3	0.43	2.9	601.8	589.1	85.5	12.7	0.40	0.04	0.9995/0.9970
SCh8T10_W30	662.2	0.39	2.7	529.8	525.2	132.4	4.6	0.38	0.06	0.9989/0.9913
SCh8T20_W30	663.7	0.42	2.7	623.8	620.9	39.9	2.9	0.41	0.02	0.9997/0.9919

SM4. Figure S2. pH time evolution of xerogels soaked in PBS

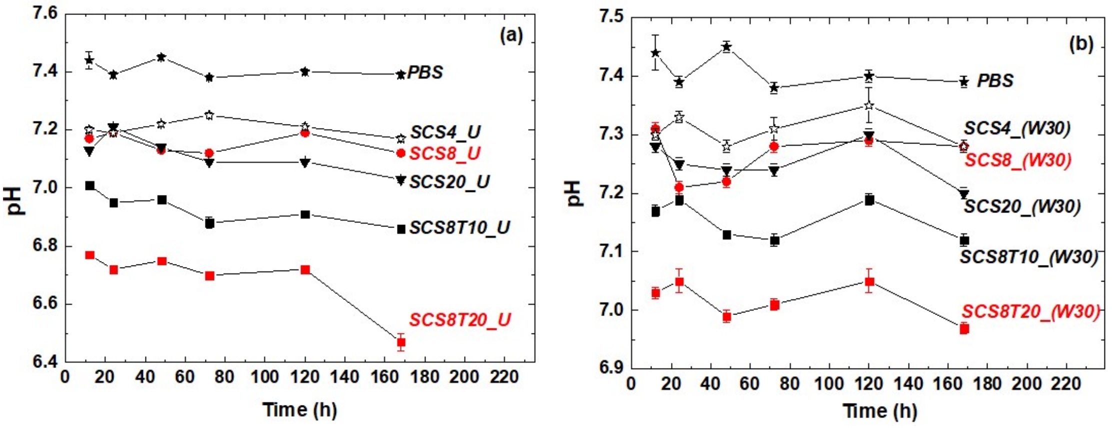

Figure S2. pH evolution of SCSx and SCSxTy xerogels soaked in PBS solution after 12, 24, 48, 72, 120 and 168 h, previously subjected to different washing treatments before evaporative drying

SM5. Figure S3. Elemental mapping at the microstructural level by scanning electron microscopy (SEM) with energy dispersive X-ray spectrometry (HA grown on SCS8T10_E7 and SCS8T20_E7 surfaces after 4 weeks soaking in SBF)

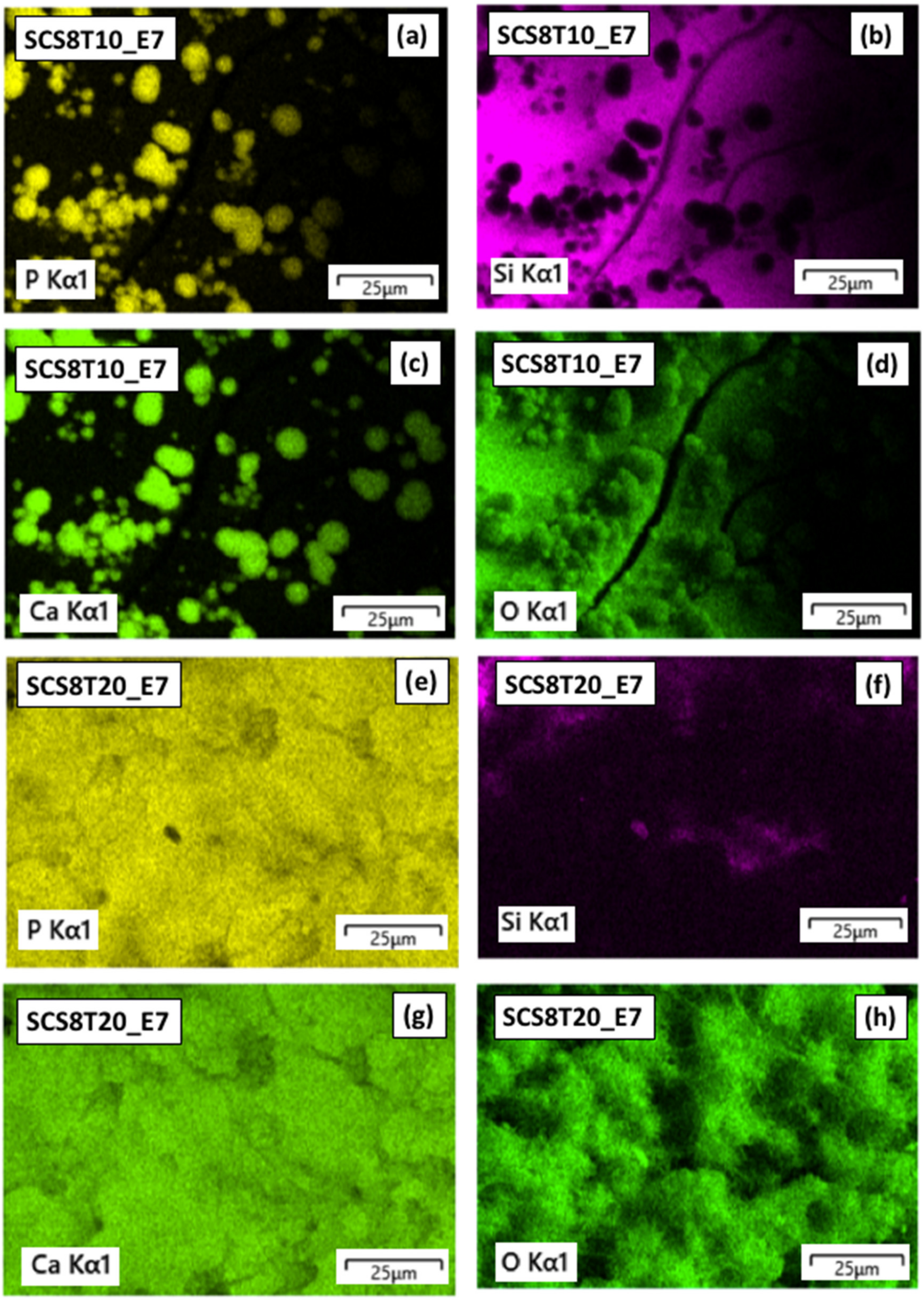

Figure S3. SEM-EDS compositional imaging of elemental distributions of the apatite layer formed after 4 weeks soaking in SBF for P (yellow), Si (purple), Ca (light green) and O (dark green) in (a, b, c, d) SCS8T10_E7 ; and (e, f, g, h) SCS8T20_E7, which surface appears almost totally covered by biomimetic apatite

SM 6: Figure S4: Live /dead assay. Positive and negative controls

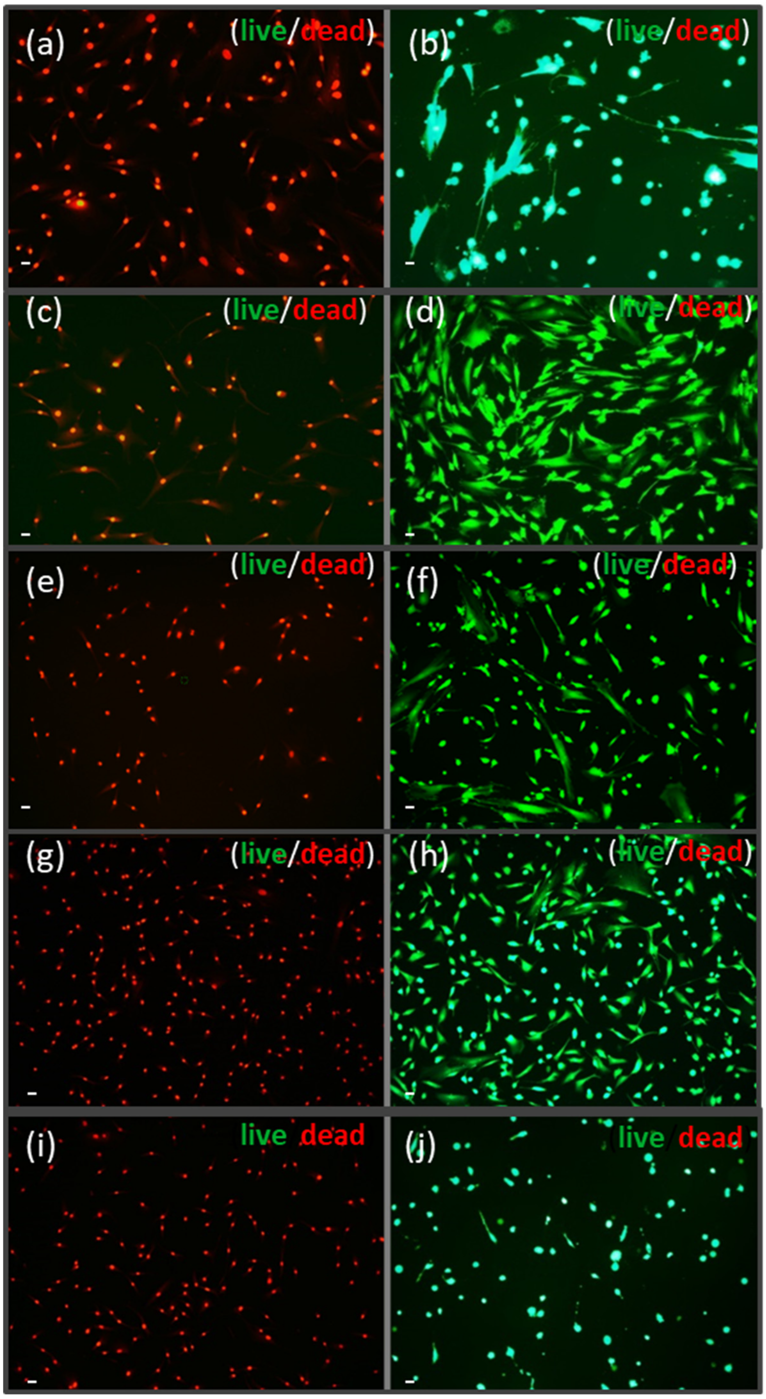

Figure S4: Negative controls after treatment with 70% ethanol for 30 minutes before staining: a, c, e, g and i. Positive controls: HOB cultures without xerogels: b, d, f, h and j. Live cells appear green; the nuclei of dead cells stained in red and Xerogels in gray. Scale bar represents 20 μm.

SM 7: Figure S5: Live /dead assay. Column graphs

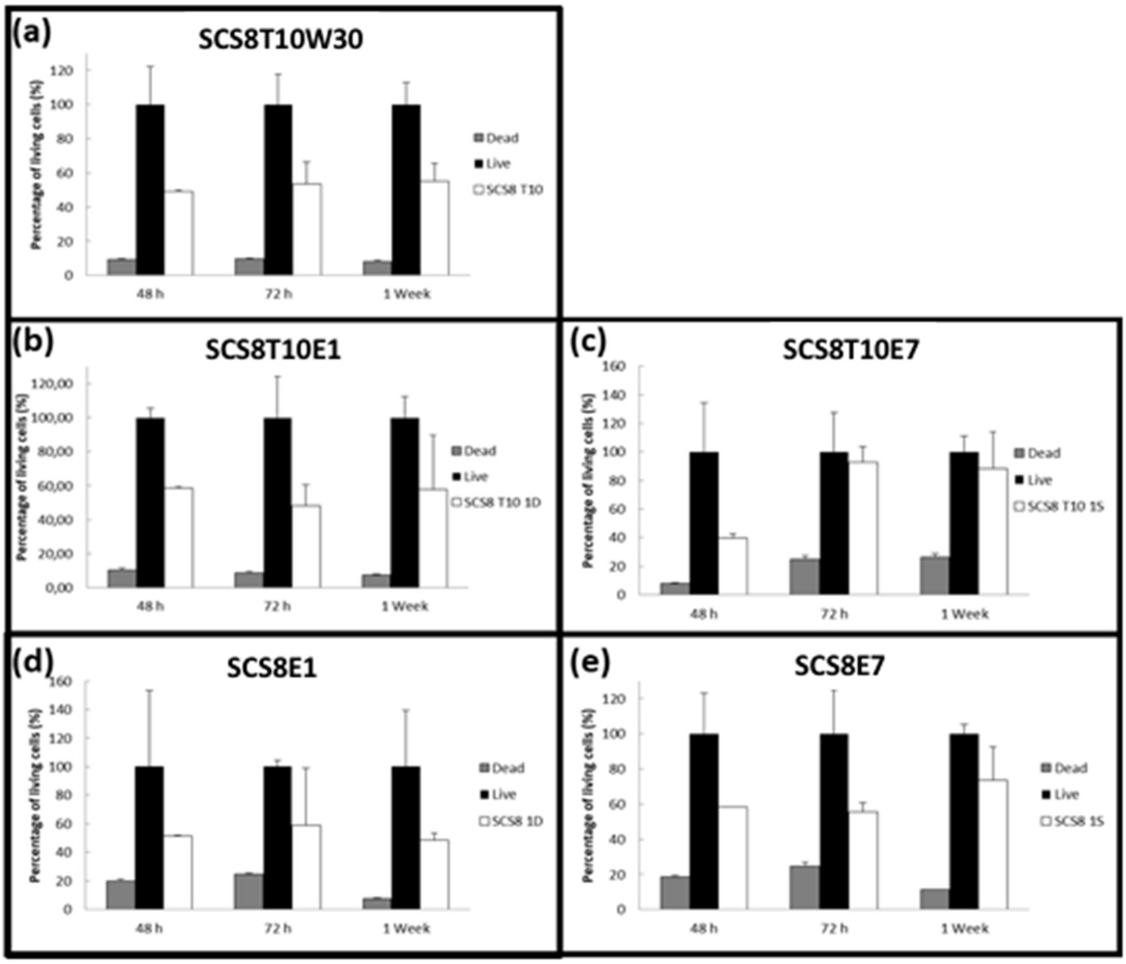

Figure S5. Percentage of live cells in HOB cultures. Dead control (Dead Control) were obtained by treating cultures with 70% methanol for 30 minutes before staining. The cultures for the reference value (100%) of live cells (Live Control) are obtained from cultures without treatments under optimal conditions for their growth at the indicated times. The cultures with the different xerogels are obtained from cultures with xerogel treatments under optimal conditions for their growth at the indicated times. Histogram representation of 48 hour, 72 hour and one week HOB cultures include negative control (Dead), positive control (Live) and treatment with (a) SCS8 T10 (b) SCS8 T10E1; (c) SCS8 T10E7; (d) SCS8E1 and (e) SCS8E7. Quantification analysed in 10 images per experiment (n=3). The values correspond to mean ± SEM. No statistically significant difference was found between the different xeromaterials (Anova test).

SM8. Table S3. Quantitative data obtained after Live Dead cell analysis. Live and dead data represent positive and negative controls respectively

	48h		72h		1 Week	
**SCS8T10W30**	Mean (%)	SD	Mean (%)	SD	Mean (%)	SD
Dead	9.851	0.077	9.821	0.159	8.075	0.860
Live	100	22.151	100	17.899	100	12.747
Sample	49.032	1.972	53.427	18.423	54.964	10.533
**SCS8T10 E1**						
Dead	10.485	0.936	8.758	0.748	7.385	0.681
Live	100	5.652	100	24.254	100	12.361
Sample	58.607	7.858	48.050	7.782	57.789	31.922
**SCS8T10 E7**						
Dead	6.259	0.468	24.617	2.759	25.994	2.770
Live	100	34.159	100	27.383	100	10.902
Sample	39.713	4.983	92.641	23.001	88.228	25.550
**SCS8E1**						
Dead	20.230	0.040	24.916	0.441	7.632	0.448
Live	100	53.485	100	4.631	100	39.645
Sample	51.561	9.985	59.176	14.727	48.796	4.745
**SCS8E7**						
Dead	20.220	0.076	24.473	2.696	11.282	0.044
Live	100	23.001	100	25.001	100	5.273
Sample	58.196	7.052	55.332	7.910	73.465	19.158

SM9. Table S4. Raw Data from table S3:

SCS8T10_U

48 hours

Negative control			Positive control			Samples		
A	B	C	A	B	C	A	B	C
1.104	1.086	1.110	7.909	12.113	27.103	6.786	3.612	4.369
1.099	1.084	1.104	7.813	16.173	31.195	8.238	2.522	4.163
1.092	1.083	1.096	9.646	12.053	27.215	4.540	6.624	6.662
1.110	1.096	1.114	7.777	4.968	15.807	9.033	5.828	3.710
1.098	1.082	1.102	16.929	14.813	29.414	6.859	3.951	8.793
1.084	1.085	1.092	10.212	20.831	37.849	6.919	7.885	6.180
1.096	1.092	1.104	4.232	30.084	40.266	1.887	5.480	5.450
1.088	1.086	1.094	8.965	9.368	24.199	3.263	9.358	3.887
1.105	1.094	1.105	6.433	6.498	20.538	5.174	6.267	5.298
1.105	1.078	1.111	5.486	7.578	21.862	3.886	3.316	3.715

72 hours

Negative control			Positive control			Samples		
A	B	C	A	B	C	A	B	C
1.000	1.004	1.000	19.729	6.297	5.953	3.649	8.405	5.147
1.000	1.000	1.000	21.392	8.861	9.101	2.712	8.922	4.277
1.000	1.000	1.000	12.597	8.169	6.800	2.926	6.418	5.163
1.000	1.000	1.102	15.303	10.372	12.653	5.552	8.014	3.486
1.000	1.000	1.051	5.440	9.427	13.254	2.912	8.721	4.439
1.000	1.000	1.000	9.172	6.803	12.991	4.029	6.902	6.756
1.000	1.095	1.000	5.362	11.356	18.274	5.233	6.957	7.937
1.010	1.000	1.000	11.141	7.487	13.792	3.159	8.101	5.471
1.000	1.021	1.180	9.124	7.619	4.738	3.141	5.873	6.429
1.000	1.000	1.000	11.838	7.786	7.350	2.413	5.472	7.104

1 week

Negative control			Positive control			Samples		
A	B	C	A	B	C	A	B	C
1.878	2.263	1.014	18.838	15.912	13.927	11.339	6.444	3.848
1.011	0.405	1.013	24.564	13.202	20.834	12.257	7.972	7.860
1.948	1.022	1.063	14.732	14.998	26.901	11.235	12.051	10.655
1.979	1.010	1.029	12.178	17.227	27.403	9.462	9.914	10.359
1.032	2.260	2.329	13.713	13.913	18.924	10.839	5.532	8.789
1.327	1.006	1.019	9.563	11.198	11.503	15.443	11.176	11.197
1.007	1.382	1.040	14.071	15.232	14.286	8.494	9.160	6.278
1.019	1.009	1.061	12.775	14.581	15.368	13.878	4.803	5.413
1.020	1.015	2.476	21.179	22.341	24.104	7.138	9.820	5.464
2.138	0.405	2.091	15.526	12.526	17.217	11.003		

SCS8T10_ E1

48 hours

Negative control			Positive control			Samples		
A	B	C	A	B	C	A	B	C
1.087	1.082	1.024	14.579	9.782	13.672	7.465	7.995	4.605
1.020	1.085	1.087	9.890	8.701	17.404	3.364	5.482	9.476
1.019	1.011	1.013	12.908	7.677	4.205	3.642	7.630	5.605
1.019	1.022	1.087	7.008	7.168	5.374	4.598	6.902	5.132
1.965	1.086	1.013	10.812	6.792	4.536	7.562	4.864	6.498
			12.252	9.529	8.538	4.370	5.793	7.801
				12.337	13.758	4.697	5.259	5.616
				11.201	17.956	6.792	11.755	5.744
				16.309	10.208			
				14.105	5.373			

72 hours

Negative control			Positive control			Samples		
A	B	C	A	B	C	A	B	C
1.016	1.017	1.018	10.348	19.793	18.096	7.727	7.426	4.821
1.015	1.021	1.021	9.098	17.146	16.562	3.671	6.962	5.111
1.014	1.017	1.842	12.815	15.049	10.053	6.316	5.886	4.747
1.017	1.018	1.021	8.618	17.754	18.304	4.725	4.960	6.229
1.020	1.833	1.023	8.285	14.631	15.411	4.572	13.628	4.688
			9.695	4.818	17.530	4.844	5.599	
			11.515	9.443	18.098	10.019	5.287	
			9.106	10.122	17.639	8.783		
			7.610	9.354	15.228			
			11.590	8.421	14.091			

1 week

Negative control			Positive control			Samples		
A	B	C	A	B	C	A	B	C
1.980	1.077	0.991	26.508	16.019	19.490	9.964	22.618	20.008
1.030	1.040	0.957	14.235	16.388	23.109	8.307	24.999	5.682
1.035	2.148	1.869	17.389	13.527	23.127	7.447	21.947	7.627
2.587	2.526	2.198	25.182	16.835	17.066	6.994	29.831	5.720
2.504	2.120	1.654	29.404	18.195	17.300	7.316	46.117	7.621
			31.632	24.462	26.296	4.240	11.961	9.825
			32.323	27.757	32.580	5.241	14.505	16.805
			31.831	29.413	30.238	6.722	13.635	9.152
					20.096	9.333	10.453	13.804
						14	22.435	17.779

SCS8T10_E7

48 hours

Negative control			Positive control			Samples		
A	B	C	A	B	C	A	B	C
0.988	0.786	0.687	7.897	15.638	9.511	4.924	3.601	5.987
0.915	0.701	0.748	8.906	13.013	7.383	6.760	2.322	7.426
0.846	0.725	0.740	14.497	17.091	7.984	9.220	3.333	2.645
0.908	0.934	0.691	27.557	20.267	9.326	5.103	3.630	6.807
1.052	1.046	0.685	19.223	22.591	6.559	5.492	2.750	3.239
0.792	0.704	0.743	22.108	12.413	7.651	5.810	4.292	4.348
0.797	0.701	0.734	11.409	23.159	6.582	4.037	7.812	2.767
0.820	0.703	0.702	4.089	20.623	6.440	9.715	7.842	4.501
0.818	0.744	0.703	10.914	12.378	12.115	4.605	6.808	4.816
0.915	1.017	1.214	9.194	15.582	10.272	3.718	6.786	

72 hours

Negative control			Positive control			Samples		
A	B	C	A	B	C	A	B	C
1.239	1.096	1.228	4.342	9.728	3.625	4.288	9.689	4.638
1.222	1.098	1.300	3.864	3.923	3.794	7.119	5.045	5.320
1.297	1.098	1.147	3.595	3.535	3.935	2.204	5.568	4.793
1.446	1.118	1.205	4.952	7.174	4.554	3.245	3.717	3.005
2.781	1.075	1.124	4.737	4.870	3.496	1.610	5.802	2.736
1.152	1.087	1.099	3.999	4.107	4.929	4.987	6.213	3.722
1.140	1.194	1.292	7.438	11.393	3.021	3.181	6.102	3.335
1.387	1.147	1.113	4.942	6.019	3.289	4.467	3.503	4.708
1.161	1.138	1.236	4.698	6.928	2.655	3.010	7.073	4.111
1.156	1.304	1.133	5.313	7.594	4.721	3.364	6.985	6.506

1 week

Negative control			Positive control			Samples		
A	B	C	A	B	C	A	B	C
1.878	2.263	1.014	6.406	4.390	3.893	11.941	4.529	3.207
1.011	0.405	1.013	7.185	3.868	3.166	14.905	3.855	3.599
1.948	1.022	1.063	5.209	3.168	2.809	6.087	2.889	3.703
1.979	1.010	1.029	3.729	3.897	6.112	4.083	2.220	3.834
1.032	2.260	2.329	2.932	3.494	8.444	3.805	3.948	4.353
1.327	1.006	1.019	4.510	3.844	8.332	2.378	3.408	3.798
1.007	1.382	1.040	4.532	3.493	6.384	3.528	3.289	3.260
1.019	1.009	1.061	5.431	5.315	7.135	3.995	4.123	3.251
1.020	1.015	2.476	6.853	7.392	2.758	4.249	5.130	5.932
2.138	0.405	2.091	9.068	6.386	4.788	5.794	5.410	

SCS8_E1

48 hours

Negative control			Positive control			Samples		
A	B	C	A	B	C	A	B	C
1.034	1.039	1.037	3.654	3.405	9.763	3.401	2.465	2.270
1.040	1.038	1.035	2.928	3.635	5.104	2.120	1.276	1.949
1.049	1.040	1.036	3.534	3.399	5.972	2.152	2.560	2.939
1.027	1.043	1.041	3.391	3.181	3.551	1.139	3.362	3.423
1.036	1.040	1.036	4.292	3.141	4.171	2.405	3.249	1.955
1.040	1.045	1.040	5.108	3.005	5.583	4.449	3.075	2.398
1.037	1.047	1.044	4.314	3.098	9.342	3.331	2.239	2.131
1.036	1.044	1.044	2.952	3.590	6.087	2.020	6.705	2.051
1.042	1.046	1.034	4.145	2.746	14.189	1.261	4.081	1.012
1.040	1.036	1.037	4.802	2.963	19.094	2.597		

72 hours

Negative control			Positive control			Samples		
A	B	C	A	B	C	A	B	C
1.154	1.814	1.936	14.415	9.780	5.469	3.719	4.408	13.372
2.161	1.967	2.364	9.814	7.341	9.555	3.859	6.259	5.616
1.148	1.017	1.000	7.085	11.425	6.010	3.061	2.874	3.923
1.214	1.886	2.031	5.799	5.447	4.223	3.541	5.697	6.555
2.540	2.089	1.891	5.485	6.494	6.556	4.740	2.620	3.732
2.577	2.412	1.911	9.153	5.766	6.253	3.850	4.601	6.166
2.469	1.906	1.978	6.292	4.897	9.931	2.979	3.318	5.576
2.420	1.990	1.874	4.879	9.407	6.494	2.224	2.999	3.177
1.148	1.794	2.545	7.506	5.525	7.133	2.329	6.710	4.027
2.422	1.819	1.127	9.348	7.589	12.109	4.378	3.508	4.617

1 week

Negative control			Positive control			Samples		
A	B	C	A	B	C	A	B	C
1.104	1.086	1.081	10.854	11.678	11.627	6.145	5.467	3.848
1.099	1.084	1.084	19.775	16.862	7.269	8.667	10.816	5.886
1.092	1.083	1.081	10.282	28.227	12.841	9.523	7.843	7.618
1.110	1.096	1.097	21.195	13.699	8.738	7.589	9.667	3.933
1.098	1.082	1.093	18.528	25.457	5.817	5.104	10.733	7.216
1.084	1.085	1.098	19.581	24.553	5.603	7.764	9.473	6.510
1.096	1.092	1.085	18.122	17.550	7.595	7.916	5.720	6.217
1.088	1.086	1.084	18.122	13.938	4.407	10.950	2.835	2.920
1.105	1.094	1.081	13.819	17.693	5.316	4.370	5.123	10.020
1.105	1.078	1.091	11.913	17.762	9.919	3.837	5.396	7.978

SCS8_ E7

48 hours

Negative control			Positive control			Samples		
A	B	C	A	B	C	A	B	C
1.114	1.109	1.139	3.436	3.677	6.276	3.017	2.676	2.319
1.157	1.125	1.131	2.766	4.319	7.093	3.354	2.792	3.752
1.127	1.128	1.108	2.992	4.336	10.866	4.049	3.047	3.655
1.170	1.108	1.142	2.789	7.684	11.910	3.084	5.692	2.309
1.119	1.142	1.126	3.253	9.131	4.874	3.858	4.989	2.957
1.160	1.125	1.121	4.249	6.830	3.657	3.315	4.386	4.152
1.126	1.144	1.132	6.195	6.954	5.149	2.216	2.921	2.660
1.130	1.144	1.123	6.650	5.865	5.368	2.410	4.083	3.609
1.121	1.137	1.143	5.373	3.648	4.131	2.485	2.789	2.004
1.129	1.127	1.108	3.505	9.001	5.749	2.275	3.141	2.873

72 hours

Negative control			Positive control			Samples		
A	B	C	A	B	C	A	B	C
1.194	1.473	1.177	5.186	4.579	3.742	1.628	3.175	2.115
1.212	1.204	1.175	7.866	4.020	3.800	1.702	2.487	1.669
1.195	1.203	1.172	6.800	3.882	3.240	1.560	4.158	3.527
1.166	0.515	1.176	6.114	4.885	3.512	2.878	3.402	2.064
1.166	1.195	1.422	2.959	3.865	3.476	3.157	2.315	2.097
1.186	1.208	1.367	6.438	3.368		3.986	4.505	2.920
1.186	1.202	1.167	7.117	5.327		2.306	1.565	2.410
1.195	1.202	1.178	4.538	5.183		1.964	2.240	2.149
0.112	1.218	1.052	6.001	3.718		1.760	3.914	1.968
0.136	1.210	1.169	4.909			2.182	1.594	2.191

1 week

Negative control			Positive control			Samples		
A	B	C	A	B	C	A	B	C
0.977	0.980	0.987	10.169	7.289	6.249	5	5.875	7.624
0.979	0.982	0.992	10.939	6.272	6.927	3.629	8.036	10.562
0.976	0.981	0.985	10.906	8.708	8.091	5.090	8.125	6.770
0.978	0.981	0.984	8.458	7.753	9.636	2.769	4.222	4.524
0.979	0.977	0.987	8.487	9.275	7.682	3.040	8.037	10.005
0.977	0.980	0.983	7.379	11.038	9.424	2.870	7.496	7.321
0.977	0.983	0.986	7.037	9.464	8.496	2.444	8.740	6.960
0.979	0.982	0.986	9.479	10.287	8.442	7.408	8.384	9.671
0.995	0.981	0.987	7.936	8.904	9.263	6.915	6.304	4.397
0.980		0.991	8.981	10.695	7.565	6.052	7.808	6.863
						3.789		

SM10. Figure S6 : Shape variables

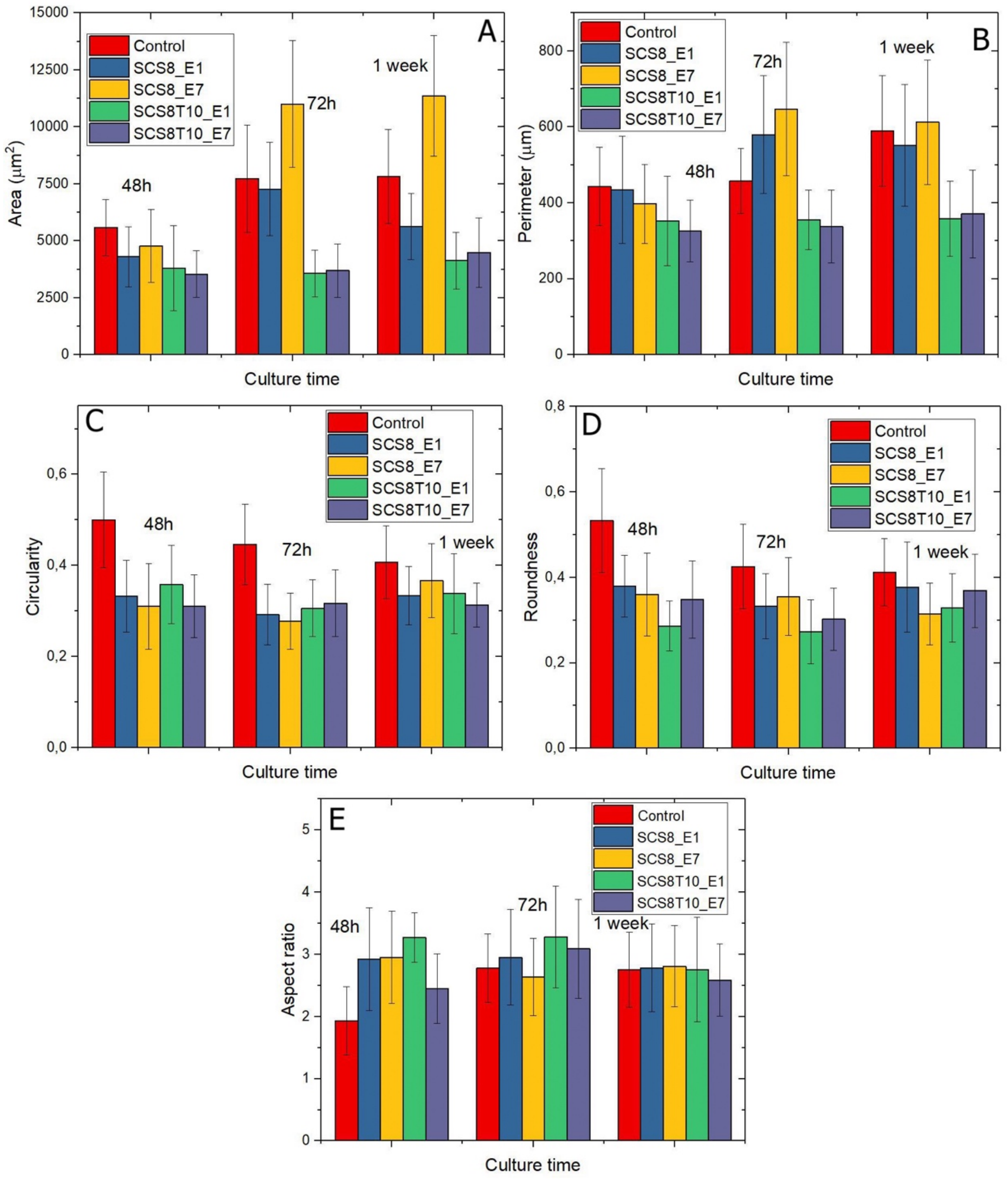

Figure S6. Shape variables. (A) Quantification of cellular spread area, (B) Quantification of cellular perimeter, (C) Quantification of cellular circularity, (D) Quantification of cellular roundness, (E) Quantification of cellular aspect ratio .

SM 11: Table S5. Significant differences between shape variables intergroups

Table S5. Shape variables analysis . CI 95%. Statystical significance p<0.005.

Variable	Significance
Area	F(14, 144) = 36.299; p<0.05
Aspect ratio	F(14, 260) = 5.943; p<0.05
Circularity	F(14, 290) = 15.798; p<0.05
Perimeter	F(14, 240) = 19.097; p<0.05
Roundness	F(14, 254) = 13.339; p<0.05

SM 12:

Table S6. Pairwise differences between groups with 95% confidence interval.

Variable	Culture time	Sample 1	Sample 2	Significance (p<0.05)
Area	48h	Control	SCS8T10_E7	t(144) = 5.31
	72h	Control	SCS8T10_E1	t(144) = 6.43
			SCS8T10_E7	t(144) = 5.99
		SCS8_E1	SCS8_E7	t(144) = 4.58
			SCS8T10_E1	t(144) = 6.93
			SCS8T10_E7	t(144) = 6.31
	7 days	Control	SCS8_E7	t(144) = 3.89
			SCS8T10_E1	t(144) = 5.71
			SCS8T10_E7	t(144) = 4.32
		SCS8_E1	SCS8_E7	t(144) = 7.52
		SCS8_E7	SCS8T10_E1	t(144) = 10.54
			SCS8T10_E7	t(144) = 8.52
Aspect ratio	48h	Control	SCS8_E1	t(260) = -5.38
			SCS8_E7	t(260) = -6.059
			SCS8T10_E1	t(260) = -9.006
		SCS8T10_E1	SCS8T10_E7	t(260) = 4.670
Circularity	48h	Control	SCS8_E1	t(290) = 6.207
			SCS8_E7	t(290) = 6.785
			SCS8T10_E1	t(290) = 4.733
			SCS8T10_E7	t(290) = 6.666
	72h	Control	SCS8_E1	t(290) = 7.777
			SCS8_E7	t(290) = 8.047
			SCS8T10_E1	t(290) = 7.000
			SCS8T10_E7	t(290) = 5.375
	7 days	Control	SCS8_E1	t(290) = 4.055
			SCS8T10_E1	t(290) = 3.631
			SCS8T10_E7	t(290) = 4.459

Perimeter	48h	Control	SCS8T10_E7	t(240) = 4.680
	72h	Control	SCS8_E7	t(240) = -4.725
			SCS8T10_E1	t(240) = 3.643
			SCS8T10_E7	t(240) = 4.285
		SCS8_E1	SCS8T10_E1	t(240) = 6.588
			SCS8T10_E7	t(240) = 6.540
		SCS8_E7	SCS8T10_E1	t(240) = 7.784
			SCS8T10_E7	t(240) = 7.536
	7 days	Control	SCS8T10_E1	t(240) = 6.969
			SCS8T10_E7	t(240) = 5.069
		SCS8_E1	SCS8T10_E1	t(240) = 5.848
			SCS8T10_E7	t(240) = 4.090
		SCS8_E7	SCS8T10_E1	t(240) = 6.658
			SCS8T10_E7	t(240) = 5.127
Roundness	48h	Control	SCS8_E1	t(254) = 5,464
			SCS8_E7	t(254) = 5,766
			SCS8T10_E1	t(254) = 8,482
			SCS8T10_E7	t(254) = 5,575
		SCS8_E1	SCS8T10_E1	t(254) = 4,650
	72h	Control	SCS8T10_E1	t(254) = 5,629
			SCS8T10_E7	t(254) = 3,967
	7 days	Control	SCS8_E7	t(254) = 4,041

SM13. Figure S7. Mineralization of SCS8T10 E1 and SCS8T10_E7

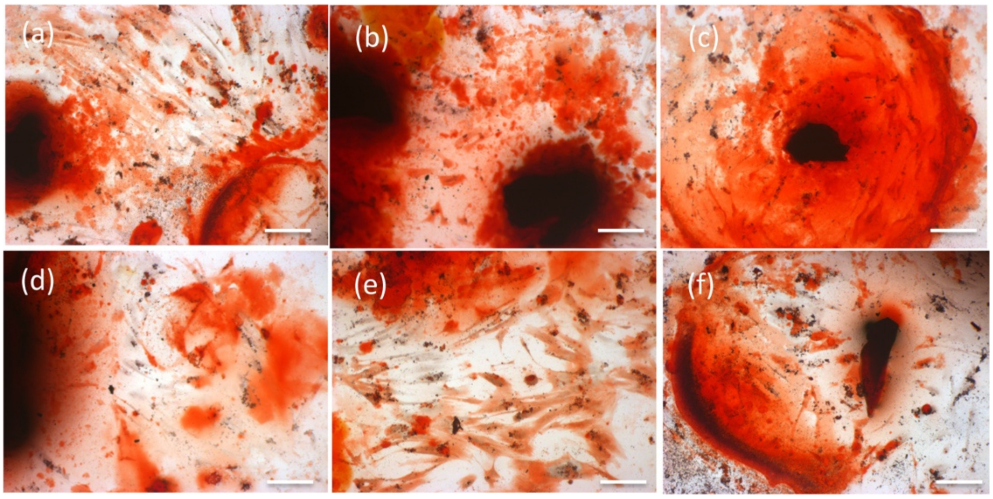

Figure S7. Alizarin red staining (red) reveals the significant effect of xerogels SCS8T10_E1and SCS8T10_E7 on mineralization. In (**A**,**B**,**C**) : HOB® cells grown on SCS8T10_E1; and in (**D**,**E**,**F**): cells grown on SCS8T10_ E7.
